# Unusual nucleosome formation and transcriptome influence by the histone H3mm18 variant

**DOI:** 10.1093/nar/gkab1137

**Published:** 2021-12-21

**Authors:** Seiya Hirai, Kosuke Tomimatsu, Atsuko Miyawaki-Kuwakado, Yoshimasa Takizawa, Tetsuro Komatsu, Taro Tachibana, Yutaro Fukushima, Yasuko Takeda, Lumi Negishi, Tomoya Kujirai, Masako Koyama, Yasuyuki Ohkawa, Hitoshi Kurumizaka

**Affiliations:** Laboratory of Chromatin Structure and Function, Institute for Quantitative Biosciences, The University of Tokyo, 1-1-1 Yayoi, Bunkyo-ku, Tokyo113-0032, Japan; Department of Biological Sciences, Graduate School of Science, The University of Tokyo, 1-1-1 Yayoi, Bunkyo-ku, Tokyo113-0032, Japan; Division of Transcriptomics, Medical Institute of Bioregulation, Kyushu University, 3-1-1 Maidashi, Higashi, Fukuoka812-0054, Japan; Division of Transcriptomics, Medical Institute of Bioregulation, Kyushu University, 3-1-1 Maidashi, Higashi, Fukuoka812-0054, Japan; Laboratory of Chromatin Structure and Function, Institute for Quantitative Biosciences, The University of Tokyo, 1-1-1 Yayoi, Bunkyo-ku, Tokyo113-0032, Japan; Institute for Molecular and Cellular Regulation, Gunma University, 3-39-15, Showa-machi, Maebashi, Gunma371-8512, Japan; Department of Bioengineering, Graduate School of Engineering, Osaka City University, Sugimoto, Sumiyoshi-ku, Osaka558-8585, Japan; Laboratory of Chromatin Structure and Function, Institute for Quantitative Biosciences, The University of Tokyo, 1-1-1 Yayoi, Bunkyo-ku, Tokyo113-0032, Japan; Department of Biological Sciences, Graduate School of Science, The University of Tokyo, 1-1-1 Yayoi, Bunkyo-ku, Tokyo113-0032, Japan; Laboratory of Chromatin Structure and Function, Institute for Quantitative Biosciences, The University of Tokyo, 1-1-1 Yayoi, Bunkyo-ku, Tokyo113-0032, Japan; Laboratory of Chromatin Structure and Function, Institute for Quantitative Biosciences, The University of Tokyo, 1-1-1 Yayoi, Bunkyo-ku, Tokyo113-0032, Japan; Laboratory of Chromatin Structure and Function, Institute for Quantitative Biosciences, The University of Tokyo, 1-1-1 Yayoi, Bunkyo-ku, Tokyo113-0032, Japan; Laboratory of Chromatin Structure and Function, Institute for Quantitative Biosciences, The University of Tokyo, 1-1-1 Yayoi, Bunkyo-ku, Tokyo113-0032, Japan; Division of Transcriptomics, Medical Institute of Bioregulation, Kyushu University, 3-1-1 Maidashi, Higashi, Fukuoka812-0054, Japan; Laboratory of Chromatin Structure and Function, Institute for Quantitative Biosciences, The University of Tokyo, 1-1-1 Yayoi, Bunkyo-ku, Tokyo113-0032, Japan; Department of Biological Sciences, Graduate School of Science, The University of Tokyo, 1-1-1 Yayoi, Bunkyo-ku, Tokyo113-0032, Japan

## Abstract

Histone H3mm18 is a non-allelic H3 variant expressed in skeletal muscle and brain in mice. However, its function has remained enigmatic. We found that H3mm18 is incorporated into chromatin in cells with low efficiency, as compared to H3.3. We determined the structures of the nucleosome core particle (NCP) containing H3mm18 by cryo-electron microscopy, which revealed that the entry/exit DNA regions are drastically disordered in the H3mm18 NCP. Consistently, the H3mm18 NCP is substantially unstable *in vitro*. The forced expression of H3mm18 in mouse myoblast C2C12 cells markedly suppressed muscle differentiation. A transcriptome analysis revealed that the forced expression of H3mm18 affected the expression of multiple genes, and suppressed a group of genes involved in muscle development. These results suggest a novel gene expression regulation system in which the chromatin landscape is altered by the formation of unusual nucleosomes with a histone variant, H3mm18, and provide important insight into understanding transcription regulation by chromatin.

## Graphical Abstract

**Graphical Abstract:**
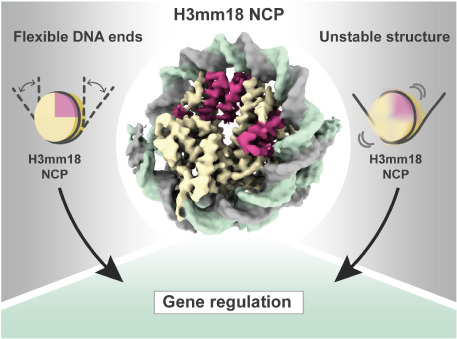
A highly mobile histone H3 variant, H3mm18, forms an unusual and unstable nucleosome with flexible DNA ends, and regulates gene expression in chromatin.

## INTRODUCTION

In eukaryotes, genomic DNA is accommodated within the nucleus, where it is compacted as chromatin (1). Histones H2A, H2B, H3 and H4 are the major protein components of chromatin, and form an elemental structural unit of chromatin termed the nucleosome (1). Histones form heterodimers, H2A–H2B and H3–H4, and two each of the H2A–H2B and H3–H4 dimers establish the histone octamer in the nucleosome (2). Approximately 150 bp of DNA are left-handedly wrapped around the histone octamer, forming the nucleosome core particle (NCP) (3).

The nucleosome is an essential architecture to compact genomic DNA, but it generally inhibits genomic DNA functions, such as transcription, replication, recombination and repair (4–6). The nucleosome-dependent gene suppression may provide more versatility of gene expression in individual tissues and cells, and play an essential role in the epigenetic regulation of genes (7–10). To accomplish this, nucleosomes must have diverse structures, stabilities, and positions in the genome (11–13).

Histone variants have from a few to 50% amino acid substitutions as compared to canonical histones, and are major factors in nucleosome diversity (14–19). Multiple copies of the canonical histone genes are encoded in the genome, and expressed in the S-phase of the cell cycle (19–24). In contrast, histone variants are encoded outside the canonical histone gene clusters, as non-allelic isoforms, and are produced in a cell-cycle independent manner (22–26). We previously identified fourteen histone H3 variants, including a testis-specific H3t variant, by the *in silico* hybridization method in mice (27). H3t is an orthologue of human H3T (27,28). The other thirteen histone H3 variants, named H3mm6–18, are evolutionally derived from H3.3 (27). H3mm7, H3mm11, H3mm12, H3mm13 and H3mm16 are efficiently incorporated into chromatin, and their distribution in genomic regions is similar to that of H3.3 (27). H3mm7 reportedly plays a central role in skeletal muscle regeneration (29). On the other hand, H3mm6, H3mm8, H3mm9, H3mm10, H3mm14, H3mm15, H3mm17 and H3mm18 have nearly homogenous distributions in nuclei, suggesting that these variants are highly mobile (27).

H3mm18 is expressed in skeletal muscle and brain (27), but its structure and function have remained elusive. In the present study, we determined the structure, characteristics, and functional significance of the H3mm18 nucleosome, and provide insights into understanding genome regulation by histone variant-mediated alterations of the chromatin architecture.

## MATERIALS AND METHODS

### Incorporation of H3mm18 within cellular chromatin

NIH3T3 cells expressing EGFP-tagged H3.3 or H3mm18 were generated as previously reported (25,29). Cells were seeded in 35-mm glass-bottom dishes (Matsunami Glass), stained with bisbenzimide H33342 fluorochrome trihydrochloride (Hoechst) (Nacalai Tesque), and imaged using a fluorescence microscope (BZ-X700; Keyence).

### Purification of histones

Mouse histones H2A, H2B, H3.3 and H4 were bacterially produced and purified by the method described previously (30). The DNA fragment encoding mouse H3mm18 was ligated into the pET15b vector at the *Nde*I-*Bam*HI sites. After transformation of *Escherichia coli* strain (BL21) cells, H3mm18 was produced as an N-terminally hexa-histidine tagged protein. The cells carrying the H3mm18 expression vector were cultured in LB medium and disrupted by sonication, and then the pellet was collected by centrifugation. The pellet was resuspended in buffer *A* (50 mM Tris–HCl, pH 8.0, 500 mM NaCl, 5% glycerol and 7 M guanidine hydrochloride), and the H3mm18 protein was recovered in the soluble fraction under denaturing conditions. The H3mm18 protein was then mixed with nickel-nitrilotriacetic acid (Ni-NTA) beads (QIAGEN) in buffer *A*, and the beads were packed into an Econo-Column (Bio-Rad). The beads were washed with buffer *B* (50 mM Tris–HCl, pH 8.0, 500 mM NaCl, 6 M urea, 5% glycerol and 10 mM imidazole), followed by buffer *B* containing 25 mM imidazole. H3mm18 was then eluted by a linear gradient of imidazole from 25 to 500 mM in buffer *B*. The hexa-histidine tag was removed by thrombin protease treatment. After the tag cleavage, 30 mM 2-mercaptoethanol was added to reduce the cysteine residues of H3mm18. The H3mm18 protein was applied to a MonoS cation exchange column, and the column was washed with buffer *C* (20 mM sodium acetate, 6 M urea, 5 mM 2-mercaptoethanol, 1 mM EDTA, and 200 mM NaCl). The H3mm18 protein was eluted by a linear gradient of 200 mM to 900 mM NaCl in buffer C. The eluted H3mm18 protein was desalted, lyophilized, and stored at 4°C. The molecular weight of the purified H3mm18 protein was confirmed by MALDI-TOF mass spectrometry (Supplementary Figure S1A). The H3.3 mutants, R40C, R53S, R40C_R53S, R72L_R83C, I124T and C-term (R128G, R129Y, R131C, G132R and R134C), were constructed by site-directed mutagenesis with the pET15b-H3.3 expression vector as the template, and prepared by the method described previously (30).

### Reconstitution and purification of NCPs

The H2A–H2B, and H3–H4 complexes were prepared as described previously (30). NCPs were reconstituted by the salt-dialysis method with H2A–H2B and H3–H4, in the presence of the 145 bp Widom 601 DNA (31), as described previously (30). The reconstituted NCPs were purified by preparative native polyacrylamide gel electrophoresis using a Prep Cell apparatus, and the buffer was exchanged to 20 mM Tris–HCl buffer (pH 7.5) containing 5% glycerol using an Amicon Ultra 30K centrifugal concentrator (Merck Millipore). The purified NCPs were stored at –80°C.

### Preparation of the PL2-6 single-chain antibody fragment

The DNA fragment encoding the light chain variable domain and heavy chain variable domain of the PL2-6 monoclonal antibody was subcloned into the pET-15b vector. In the resulting PL2-6 single-chain antibody variable fragment (scFv), the light chain variable domain and heavy chain variable domain were connected with a (GGGGS)_3_ linker peptide, as described previously (32). Expression and purification of PL2-6 scFv were performed according to the method described previously (33). PL2-6 scFv was expressed in *Escherichia coli* BL21-CodonPlus(DE3)-RIPL cells by induction with isopropyl β-d-1-thiogalactopyranoside (200 nM). The cells were harvested and disrupted by sonication. The PL2-6 scFv peptide was obtained in the insoluble fraction, and recovered in 100 mM Tris–HCl buffer (pH 8.0), containing 2 mM EDTA, 1 mM dithiothreitol and 6M guanidine hydrochloride. After centrifugation, the supernatant was concentrated to 10 ml, and added to 1,000 ml of refolding buffer, containing 100 mM Tris–HCl buffer (pH 9.5), 1 mM EDTA, and 0.5 M arginine. The refolding solution containing the PL2-6 scFv peptide was adjusted to pH 9.5 by adding HCl, and oxidized glutathione (final concentration 551 mg/ml) was added. The resulting solution was incubated at 4°C for 48 h, and then dialyzed twice against 20 mM Tris–HCl buffer (pH 7.5) containing 113 mM urea for 16 h. After dialysis, the pH value of the sample solution was adjusted to 7.8 by adding HCl. The supernatant was obtained by centrifugation, and then clarified by passage through a 0.45 μm filter (Thermo Fisher Scientific). The resulting soluble fraction containing the PL2-6 scFv peptide was mixed with SP Sepharose Fast Flow resin (4 ml) (GE Healthcare). The resin bound to the PL2-6 scFv peptide was packed into an Econo-Column (Bio-Rad), and was washed with 150 ml of 20 mM Tris–HCl buffer (pH 7.5), followed by 20 ml of 20 mM Tris–HCl buffer (pH 7.5) containing 100 mM NaCl. The PL2-6 scFv peptide was eluted with 20 mM Tris–HCl buffer (pH 7.4) containing 300 mM NaCl. The resulting PL2-6 scFv peptide was mixed with 5 ml of nickel-nitrilotriacetic acid (Ni-NTA) beads (QIAGEN), and the slurry was packed into an Econo-Column (Bio-Rad). The beads were washed with Ni buffer (20 mM TrisvHCl (pH 7.4), 300 mM NaCl, and 40 mM imidazole). The PL2-6 scFv peptide was then eluted by a linear gradient of imidazole from 40 to 250 mM in Ni buffer. The PL2-6 scFv peptide was further purified by gel filtration chromatography on a Hiload 16/600 Superdex 75 (GE Healthcare) column equilibrated with 20 mM Tris–HCl buffer (pH 7.4), containing 150 mM NaCl and 1 mM EDTA. The purified PL2-6 scFv peptide was flash-frozen in liquid nitrogen and stored at –80°C.

### Structure analysis by cryo-EM

The purified nucleosome containing H3mm18 was fixed by the gradient fixation (GraFix) method (34). The sucrose and paraformaldehyde gradient solution was prepared with low sucrose concentration buffer (10 mM HEPES–NaOH (pH 7.5), 20 mM NaCl, 1 mM dithiothreitol and 5% sucrose) and high sucrose concentration buffer (10 mM HEPES–NaOH (pH 7.5), 20 mM NaCl, 1 mM dithiothreitol, 20% sucrose and 4% paraformaldehyde), using a gradient master instrument (SKB). The NCP sample (0.4 nmol) was loaded on the top of the gradient solution, and fractionated by centrifugation at 27 000 rpm at 4°C for 16 h, using a Beckman SW41 rotor. The fractions containing the NCP were collected and desalted on a PD-10 column (GE Healthcare) equilibrated with 20 mM HEPES–KOH (pH 7.5) buffer, containing 50 mM potassium acetate, 0.2 μM zinc acetate, and 0.1 mM Tris(2-carboxyethyl)phosphine. The sample was then concentrated to 10.6 μM by an Amicon Ultra 30K centrifugal concentrator (Merck Millipore), and was plunge frozen by vitrification using a Vitrobot Mark IV (Thermo Fisher Scientific) on a Quantifoil R1.2/1.3 copper grid, which had been glow discharged for 1 min by a PIB-10 Bombarder (Vacuum Device Inc.). For the H3mm18 NCP with PL2-6 scFv, the H3mm18 NCP and PL2-6 scFv were mixed at a 1:4 molar ratio in reaction solution (20 mM Tris–HCl (pH 7.5), 34.5 mM NaCl, 0.858% glycerol, 230 μM EDTA and 770 μM dithiothreitol). The reaction mixture was incubated at 25°C for 30 min, and then plunge frozen by vitrification using a Vitrobot Mark IV (Thermo Fisher Scientific) on an UltraAufoil R1.2/1.3 300 mesh grid, which had been plasma cleaned for 30 s by a Solarus II (Gatan).

### Cryo-EM data collection

Cryo-EM data of the crosslinked H3mm18 NCP prepared by GraFix and the non-crosslinked H3mm18 NCP with PL2-6 scFv were collected using the SerialEM automation software (35) on KriosG3i and KriosG4 microscopes (Thermo Fisher Scientific), operating at 300 kV at a nominal magnification of 81 000× (pixel sizes of 1.1 and 1.06 Å), with defocus ranging from −1.0 to −2.5 μm, respectively. Digital micrographs of the crosslinked H3mm18 NCP and the non-crosslinked H3mm18 NCP were recorded with 5.6 and 4.5 s exposure times on a K3 BioQuantum direct detection camera (Gatan) in the energy-filter TEM mode with a 25 eV slit, at a total dose of ∼57 electrons per Å^2^ with a total of 40 frames, respectively.

### Image processing

In total, 10 006 movies for the crosslinked H3mm18 NCP, and 4914 movies for the non-crosslinked H3mm18 NCP were aligned using MOTIONCOR2 (36), with dose weighting. The estimation of the contrast transfer function (CTF) was performed by CTFFIND4 (37) from digital micrographs with dose weighting. RELION 3.1 (38) was used for all subsequent image processing. In total, 3 584 929 particles of the crosslinked H3mm18 NCP, and 790 901 particles of the non-crosslinked H3mm18 NCP were picked automatically, followed by 2D classification to remove junk particles, resulting in the selection of 2 317 022 particles of the crosslinked H3mm18 NCP, and 629 528 particles of the non-crosslinked H3mm18 NCP. For the crosslinked H3mm18 NCP, the crystal structure of a canonical NCP (PDB: 3LZ0, (39)) was low-pass filtered to 60 Å, and used as an initial reference model. After the first round of 3D classification, 274 546 particles were selected for 3D refinement, followed by particle polishing and CTF refinement. The final map of the crosslinked H3mm18 NCP was sharpened with a *B*-factor (−51.3 Å^2^). For the non-crosslinked H3mm18 NCP, the *ab initio* model generated by Relion was low-pass filtered to 60 Å, and used as the initial reference model. After the first round of 3D classification, 224 465 particles were selected for 3D refinement, followed by particle polishing and CTF refinement. The final map of the non-crosslinked H3mm18 NCP was sharpened with a *B*-factor (−149.9 Å^2^). The resolutions of the final 3D maps were estimated by the gold standard Fourier Shell Correlation (FSC) at FSC = 0.143 (40), and normalized with MAPMAN (41). The local resolution maps were calculated by RELION 3.1, and visualized with UCSF Chimera (42). The details of the processing statistics for the crosslinked H3mm18 NCP and the non-crosslinked H3mm18 NCP are shown in Table 1.

**Table 1.** Cryo-EM data collection, processing, refinement and validation statistics

Sample	H3mm18 nucleosome (EMD-30631) (PDB 7DBH)	H3mm18 nucleosome with PL2-6 (EMD-31882) (PDB 7VBM)
**Data collection**		
Electron microscope	KriosG3i	KriosG4
Camera	K3	K3
Pixel size (Å/pix)	1.1	1.06
Defocus range (μm)	-1.0 to -2.5	-1.0 to -2.5
Exposure time (second)	5.6	4.5
Total dose (e/Å^2^)	57	57
Movie frames (no.)	40	40
Total micrographs (no.)	10 006	4914
**Reconstruction**		
Software	Relion 3.1	Relion 3.1
Particles for 2D classification	3 584 929	790 901
Particles for 3D classification	2 317 022	629 528
Particles in the final map (no.)	274 546	224 465
Symmetry	C1	C1
Final resolution (Å)	3.6	3.4
FSC threshold	0.143	0.143
Map sharpening *B*factor (Å^2^)	-51.3	-149.9
**Model building**		
Software	Coot	Coot
**Refinement**		
Software	Phenix	Phenix, ISOLDE
**Model composition**		
Protein	697	677
Nucleotide	252	252
**Validation**		
MolProbity score	1.78	0.99
Clash score	11.67	2.18
R.m.s. deviations		
Bond lengths (Å)	0.007	0.006
Bond angles (°)	0.851	0.911
**Ramachandran plot**		
Favored (%)	96.77	98.03
Allowed (%)	3.23	1.97
Outliers (%)	0	0

### Model building

The atomic model of the crosslinked H3mm18 NCP was built based on the crystal structure of the *Xenopus laevis* NCP containing the Widom 601 positioning sequence (PDB ID: 3LZ0, (39)). The atomic coordinates of the *X. laevis* NCP were fitted to the cryo-EM map of the H3mm18 NCP by the *Cryo_fit* program (43). The amino acid residues of the histones were adjusted into the mouse histones. The resulting atomic model was refined using phenix_real_space_refine (44) against the cryo-EM map, and edited manually with COOT (45). For the non-crosslinked H3mm18 NCP structure aided by PL2-6 scFv, the final model of the crosslinked H3mm18 NCP structure was used as the starting model. The model was refined by phenix_real_space_refine (44) and manually rebuilt using interactive molecular dynamics flexible fitting with the ISOLDE software (46). The final models of the crosslinked and non-crosslinked H3mm18 NCP structures were validated by the MolProbity program ((47); Table 1). Structural figures were prepared with PyMOL (The PyMOL Molecular Graphics System, Version 2.0, Schrödinger, LLC.), and ChimeraX (48).

### Thermal stability assay

The NCP samples (2.25 μg for DNA) were subjected to a thermal stability assay. The assay was performed in 19 μl of reaction solution (17 mM Tris–HCl (pH 7.5), 0.85 mM dithiothreitol, 4.25% glycerol, 100 mM NaCl and 5× SYPRO Orange (SIGMA-Aldrich)) as described previously (49). The samples were tested with a temperature gradient from 25°C to 95°C in steps of 1°C/min. The fluorescence signal of the SYPRO Orange was detected by a Step One Plus real-time PCR instrument (Applied Biosystems). Normalization of the signal was performed as follows: *F*(*T*)_normalized_ = [*F*(*T*) – *F*(26)]/[*F*(95) – *F*(26)], where *F*(*T*) indicates the fluorescence signal intensity at a particular temperature.

### MNase assay

The NCP samples (1.4 μg) were subjected to the MNase assay in 70 μl of reaction solution (50 mM Tris–HCl (pH 8.0), 1.9 mM dithiothreitol, 2.5% glycerol, 25 mM NaCl and 2.5 mM CaCl_2_). After an incubation at 37°C for the indicated time, each aliquot (10 μl) was taken and mixed with 5 μl of the reaction stop solution (20 mM Tris–HCl (pH 8.0), 20 mM EDTA, 0.1% SDS, and 0.48 mg/ml proteinase K (SIGMA-Aldrich)). The resulting DNA fragments were analyzed by native-polyacrylamide gel electrophoresis with ethidium bromide staining. The gel images were obtained with an Amersham imager (GE Healthcare).

### Plasmids for differentiation assay and transcriptome analysis

The expression vectors for the N-terminally EGFP-tagged histones H3.3 and H3mm18 were described previously (27), and are based on the Tet Expression Vector pT2A-TRETIBI bidirectionally expressing EGFP-fused proteins and *NeoR*. The expression vectors for C-terminally EGFP-tagged histone H3.3 and H3mm18 were also constructed with a similar subcloning strategy, except that the *Puro* expression cassette was inserted instead of *NeoR*. To generate stably expressing cells, these plasmids were transfected together with pCAGGS-TP and pT2A-CAG-rtTA2S-M2, as described previously (27). For the *H3mm18* reporter constructs, using genomic DNA extracted from mouse tails as the template, the corresponding genomic regions were PCR amplified with a common forward primer (5′-AAAGAATTCTGTTCGGAGCCTTCGCAGC-3′) and specific reverse primers as follows: 5′-CCCGGATCCTTAAGCACATTCTCTGCATATG-3′, 5′-CCCGGATCCATGTATTTTAATAGCAAACTTACAGG-3′, 5′-AAAGGATCCCCTTCATTACCCTCTGGTCC-3′, and 5′-CCCCTGCAGTTAAGCACATTCTCTGCATATG-3′ for ΔUTR-GA, ΔGA, H3mm18 and 18-pA, respectively. These fragments were inserted into the pT2A-TRETIBI/EGFP-H3.3 vector, previously digested with either EcoRI and BglII or EcoRI and PstI.

### Cells

C2C12 and NIH3T3 cells were purchased from the American Type Culture Collection (ATCC). C2C12 cells were grown in Dulbecco's modified Eagle's medium (DMEM) supplemented with 20% fetal bovine serum. For differentiation, cells were transferred to DMEM containing 2% horse serum upon reaching confluence. NIH3T3 cells were grown in DMEM supplemented with 10% calf serum. Introduction of MyoD into NIH3T3 cells was accomplished using retroviral vectors, as described previously (29).

The H3mm18 knockout (KO) C2C12 cells were generated by the electroporation of reconstituted Cas9-crRNA-tracrRNA ribonucleoprotein complexes, according to IDT’s protocol. Briefly, equimolar concentrations of Alt-R CRISPR-Cas9 cRNA and Alt-R CRISPR-Cas9 tracrRNA (IDT) were heated at 95°C for 5 min, followed by cooling to RT for duplex formation. The ribonucleoprotein complexes were formed by mixing crRNA:tracrRNA duplex and Alt-R S.p. HiFi Cas9 Nuclease (IDT), and electroporated into cells using a Neon Transfection System (Invitrogen) according to the manufacturer's instructions. On the following day, the cells were single-cell sorted into 96-well plates, using a fluorescence-activated cell sorter (SH800, Sony), for clonal isolation. We designed four crRNAs targeting the *H3mm18* gene, two for upstream (5′-GAGCTGTCTCACCTAGATGT-3′ and 5′-ATATAGCCACACTCGGACAC-3′), and two for downstream (5′-TGTGCTAAGAAAGCCCGGTA-3′ and 5′-GGTTTATTAAGACCCAACGA-3′), which were introduced together into the cells in a single electroporation.

Plasmid transfection was performed using the Lipofectamine 2000 Transfection Reagent (Thermo Fisher Scientific) according to the manufacturer's instructions. For transient transfection, cells were collected at 24 h post transfection. To generate stable transfectants, cells were cultured for 14–21 days in the presence of 1 μg/ml of puromycin or 1 mg/mL G418, together with 1 μg/ml doxycycline (Dox) for the Tet-On system. EGFP-positive cells were selected by the cell sorter.

### Quantitative RT-PCR (RT-qPCR)

Total RNA was extracted from cells using Sepasol-RNA I Super G (Nacalai Tesque) and reverse-transcribed with PrimeScript II Reverse Transcriptase (Takara) and either an oligo-dT primer or a random 6-mer, according to the manufacturers’ protocols. Real-time PCR amplification and detection were performed using Thunderbird qPCR Mix (Toyobo) in the PikoReal Real-Time PCR System (Thermo Fisher Scientific) under the following thermocycling conditions: 30 sec at 95°C, 45 cycles of 5 s at 95°C, 30 s at 65°C and 20 s at 72°C. The primers used are as follows: 5′-CTCAGGACTTCAAAACAGATCTGT-3′ and 5′-CGCATATGCGGCCTGCTAGTTGGG-3′ for *H3mm18*, 5′-ATCAGCCATGATGGATACTTTCTC-3′ and 5′-GTTCTTTTTGTCAAGACCGACCT-3′ for *NeoR*, and 5′-CTCTGACTACCCTCCACTTGGTCG-3′ and 5′-ATTAAGACTGGGGTGGCAGGTGTT-3′ for *Eef1a1*, 5′-GACTTCAACAGCAACTCCCACTCT-3′ and 5′- GGTTTCTTACTCCTTGGAGGCCAT-3′ for *Gapdh*, 5′-AAGTCCAATCATTGGGCTCTGTCC-3′ and 5′-ACGGACTTTTATTTAAGGCAGGGC-3′ for *Ckm*, 5′-TTGTGCACCGCAAATGCTTCTAGG-3′ and 5′-ATGTACACGTCAAAAACAGGCGCC-3′ for *Acta1*, and 5′-AAAGCCATCACTTCTGTAGCAGGG-3′ and 5′-TCTCTGGACTCCATCTTTCTCTCC-3′ for *Myog*.

### Generation of antibodies against H3mm18

Rat monoclonal antibodies against H3mm18 were generated. The specific peptide (KQTARKSTGDKAPR, amino acids 4–17) was synthesized for use as an antigen (Eurofins). Hybridoma cells were isolated by limiting dilution and screened using culture supernatants in ICC on NIH3T3 cells expressing either EGFP-tagged H3mm18 or H3.3 (as a control). Positive clones were single-cell isolated by the cell sorter and again screened as above. Specificities of antibodies were assessed by immunoblotting and immunocytochemistry. Clone 1C8-7C11 was used in this study.

### Immunocytochemistry (ICC)

ICC was performed as described previously (29), with the following modifications. Cells were grown in ibidi 24-well plates (μ-Plate 24 Well Black) or on round cover glasses (Matsunami), and fixed with 4% paraformaldehyde in PBS for 10 min at RT. Cells were visualized with a Keyence BZ-X710 microscope system. Mouse anti-MyHC (eBioscience, MF20, 1:1000) was used.

### Biochemical fractionation and immunoprecipitation (IP)

Biochemical fractionations for preparing chromatin-unbound and chromatin fractionations were performed essentially as described previously (50). Briefly, cells were suspended in low salt extraction buffer (20 mM Tris–HCl [pH 7.5], 100 mM KCl, 0.4 mM EDTA, 0.1% Triton X-100, 10% glycerol, 1 mM β-mercaptoethanol) supplemented with a cOmplete ULTRA mini Protease Inhibitor Cocktail Tablet (Roche). After Dounce homogenization, the soluble chromatin-unbound fraction was separated by centrifugation at 14 000 × *g* at 4°C for 30 min, using a tabletop centrifuge. The insoluble pellet was then suspended in High salt extraction buffer (20 mM HEPES–KOH [pH 7.4], 400 mM KCl, 5 mM MgCl_2_, 0.1% Tween 20, 10% glycerol, 1 mM β-mercaptoethanol) supplemented with a cOmplete ULTRA mini Protease Inhibitor Cocktail Tablet, and subjected to brief sonication. The solubilized chromatin fraction was collected after centrifugation at top speed at 4°C for 30 min, using a tabletop centrifuge. For IP experiments, antibodies (mouse anti-GFP (clone 34F6, KISHIDA, 2 μg per sample), rabbit anti-DDDDK/FLAG (MBL, PM020, 1 μl per sample), and rat anti-H3mm18 (clone 1C8-7C11, 2 μg per sample)) were mixed with Dynabeads Protein G (Invitrogen) in PBS-T (0.05% Tween 20 in PBS) and rotated at 4°C for 1 hr. After washing with PBS-T, the beads were added to the chromatin-unbound fractions prepared as described above, and rotated at 4°C for 2 h. The beads were then washed three times with ice-cold Low salt extraction buffer. Finally, elution buffer (100 mM glycine–HCl [pH 2.5], 150 mM NaCl) was added to the beads to obtain the precipitated complexes, followed by neutralization with 1 M Tris–HCl [pH 8.0]. The following western blotting was performed as described previously (29). Antibodies used for immunoblotting were as follows: rabbit anti-Hsp90 (Santa Cruz, H-114, 1:2000), rabbit anti-GFP (BioAcademia, 60-011, 1:1000), mouse anti-GFP (Nacalai, GF200, 1:500), rat anti-H3mm18 (clone 1C8-7C11, 2 mg/ml, 1:1000), mouse anti-MyHC (eBioscience, MF20, 1:1000), rabbit anti-Myogenin (Santa Cruz, M-225, 1:100), rat anti-MyoD (KISHIDA, 5F11, 1:1000), rabbit anti-DDDDK/FLAG (MBL, PM020, 1:1000), and rabbit anti-DAXX (Santa Cruz, M-112, 1:1500).

### Transcriptome analysis using CEL-Seq2

CEL-Seq2 for C2C12 samples was carried out as described previously (29,51). Sequencing was performed using a HiSeq PE Rapid Cluster Kit v2 (Illumina) in an Illumina HiSeq 1500 system with 15 cycles for read 1 and 36 cycles for read 2. The extraction of sample barcodes and unique-molecular-identifiers (UMIs) was performed using UMItools (version 1.0.1) (52) with the command “umi_tools extract -I r1.fastq —read2-in = r2.fastq —bc-pattern = NNNNNNCCCCCC —read2-stdout”. The reads were trimmed using the Cutadapt (version 2.6) wrapper, Trim Galore (version 0.6.6), with the option “-a GATCGTCGGACT”. The reads were mapped to the reference genome (GRCm38, pre-built HISAT2 index, genome_snp_tran) using the aligning software HISAT2 (version 2.1.0) (53–55). The featureCounts software (version 2.0.1) (56) was used to obtain read counts per gene. Note that UMIs were not used and mitochondrial genes were excluded in the following analysis. The DESeq2 software (1.28.1) (57) was used to obtain the normalized counts and log_2_ Fold changes and to extract DEGs that met Benjamini and Hochberg corrected Wald *P* < 0.1. In the MA plots, the means of normalized counts show the average expression levels for samples used in the log_2_ Fold changes. Genes with log_2_ Fold changes over 5 and under −5 were plotted in triangles at 5 and −5, respectively in all MA plots. The Gene Ontology (GO) analysis was performed using R package clusterProfiler (version 3.16.1) (58) with the settings: OrgDb = “org.Mm.eg.db”, keyType = ‘ENSEMBL’, ont = “BP”, pAdjustMethod = “BH”, pvalueCutoff = 0.1, qvalueCutoff = 1.0. The gene ratio is the percentage of the genes in the GO term in the identified genes among the cluster. The heatmap shows the row (gene)-wise *Z* scores for regularized variance-stabilizing transformed counts (calculated by vst function in DESeq2) of the selected genes.

### Transcriptome analysis using BRB-Seq

BRB-Seq was performed as described previously (59), except that the cDNA was synthesized with RT primers for CEL-Seq2 (51) instead of those described in the original BRB-Seq protocol. Sequencing was performed using a HiSeq PE Rapid Cluster Kit v2 (Illumina) in an Illumina HiSeq 1500 system with the following cycles: 15 cycles for read 1 and 40 cycles for read 2 for NIH3T3 samples.

The extraction of sample barcodes and unique-molecular-identifiers (UMIs) was performed using UMI-tools (version 1.0.1) (52) with the following commands: umi_tools extract -I r1.fastq —read2-in = r2.fastq —bc-pattern = NNNNNNCCCCCCCCC —read2-stdout. The reads were trimmed using the Cutadapt (version 2.6) wrapper Trim Galore (version 0.6.6), with the option -a GATCGTCGGACT. The reads were mapped by the aligning software HISAT2 (version 2.1.0) (55) to the reference genome (GRCm38, pre-built HISAT2 index, genome_snp_tran). The mapped reads were sorted by SAMtools (version 1.9-170) (60). Read counts per gene were obtained using featureCounts (version 2.0.1) (56). Note that UMIs were not used and mitochondrial chromosome genes were excluded in the following analysis. The DESeq2 software (1.28.1) (57) was used to calculate normalized counts and log_2_ fold changes and to extract DEGs that met the following criteria: Benjamini and Hochberg corrected Wald *P* < 0.1. Means of normalized counts in MA plots are the average expression levels for samples used in log_2_ fold changes. Genes with log_2_ fold changes over 5 and under −5 were plotted with triangles at 5 and −5, respectively, in all MA plots.

### Chromatin profiling using ATAC-Seq

ATAC-Seq was performed as described previously (29). Sequencing was performed with an Illumina NovaSeq 6000 system with 51 cycles for both read 1 and read 2, using the following kits (Illumina): NovaSeq 6000 S2 Reagent Kit for ATAC-Seq.

Raw sequencing data were trimmed using the Cutadapt (version 2.6) (61) wrapper Trim Galore (version 0.6.6), with the option —2colour 20. The reads were mapped by the aligning software Bowtie2 (version 2.3.5.1) (62) to the reference genome (UCSC mm10, Bowtie 2 index), and the uniquely mapped reads were retained for the following analyses. The mapped reads were sorted by SAMtools (version 1.9-170) (60). For the visualization of ATAC-Seq signals on the Integrative Genomics Viewer (bigwig), read counts of 100 bp bins in the genome were normalized as counts per million (CPM) and counts of each bin were smoothed with a 1000 bp window by the deepTools software (version 3.3.0) (63) with the options: bamCoverage —binSize 100 —normalizeUsing CPM —smoothLength 1000. The bedtools software (version 2.29.2) (64) with the “multicov” option was used to count the ATAC-Seq reads within 5 kb ± TSS regions of the first exons. The DESeq2 software (1.28.1) (57) was used to calculate log_2_ fold changes for each 5 kb ± TSS region.

For the plots of fold changes of gene expression and ATAC peak signals on the TSS, genes with means of normalized counts of BRB-Seq at each time point that were over 50 (colored blue) were used. Black dots indicate the 54 downregulated DEGs at 48 hours of differentiation. The 2D kernel density of the total genes (colored blue) was drawn by the stat_density2d function in ggplot2. The 95% confidence ellipses of the 54 downregulated DEGs at 48 hours (red) and total genes (blue) were drawn by the stat_ellipse function.

## RESULTS

### H3mm18 is incorporated into chromatin with low efficiency in cells

**Figure 1.**
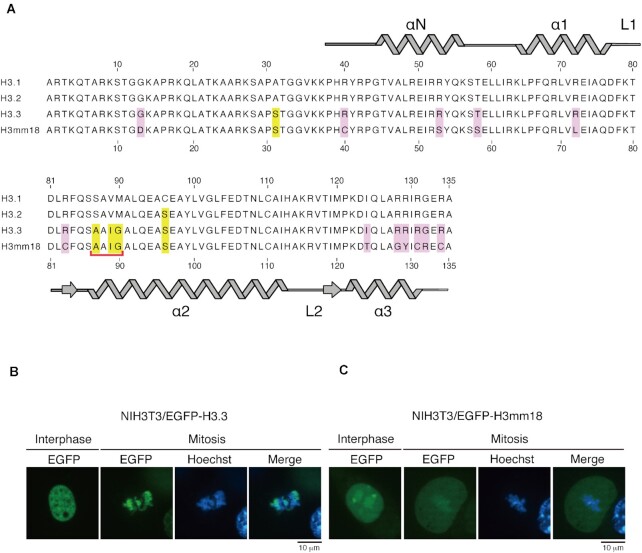
H3mm18 is incorporated into chromatin in cells. (**A**) Amino acid sequence alignment of H3.3 and H3mm18. The conserved residues between H3.3 and H3mm18 are represented with yellow boxes, and the H3mm18-specific residues are represented with pink boxes. The chaperone-recognition motif conserved between H3.3 and H3mm18 is marked by a red bracket. (**B**, **C**) NIH3T3 cells producing EGFP-tagged H3.3 (B) or H3mm18 (C). Genomic DNA was visualized by staining with bisbenzimide H33342 fluorochrome trihydrochloride (Hoechst).

H3mm18 contains 12 amino acid differences, at positions 13, 40, 53, 58, 72, 83, 124, 128, 129, 131, 132 and 134, as compared to H3.3. The Asp13, Cys40, Ser53, Ser58, Leu72, Cys83, Thr124, Gly128, Tyr129, Cys131, Arg132 and Cys134 residues of H3mm18 correspond to the Gly13, Arg40, Arg53, Thr58, Arg72, Arg83, Ile124, Arg128, Arg129, Arg131, Gly132 and Arg134 residues of H3.3, respectively (Figure 1A). These substitutions replace many basic Arg residues of H3.3 with neutral residues in H3mm18. The Arg residues play a key role in the histone-DNA interactions in the nucleosome (3).

To test whether H3mm18 is incorporated into chromatin in cells, EGFP-tagged H3mm18 or H3.3 was expressed in NIH3T3 cells. In interphase cells, both EGFP-tagged H3.3 and H3mm18 were localized in the nucleus (Figure 1B, C). In mitotic cells, the EGFP-tagged H3.3 substantially accumulated in the region that overlapped with the genomic DNA stained with bisbenzimide H33342 fluorochrome trihydrochloride (Hoechst), indicating that H3.3 is efficiently incorporated into chromatin in cells (Figure 1B). Interestingly, the chromatin incorporation of EGFP-tagged H3mm18 was also observed in mitotic cells, although its efficiency was low as compared to EGFP-tagged H3.3 (Figure 1C). Therefore, H3mm18 has the potential to be incorporated into chromatin in cells.

### The cryo-EM structure of the NCP containing H3mm18

To study the structural consequences of the H3mm18 incorporation into nucleosomes, we purified H3mm18 as a recombinant protein. The molecular weight of the recombinant H3mm18 protein was confirmed by MALDI-TOF mass spectrometry (Supplementary Figure S1A). The purified H3mm18 migrated faster than H3.3 in a sodium dodecyl sulfate polyacrylamide gel electrophoresis (SDS-PAGE) analysis (Supplementary Figure S1B). H3mm18 formed a nucleosome core particle (NCP) with histones H2A, H2B, H4, and the 145 bp Widom 601 DNA (31,65). The migration of the H3mm18 NCP in a native polyacrylamide gel electrophoresis analysis was clearly slower than that of the H3.3 NCP (Supplementary Figure S1C). This suggested that structure and/or physical properties of the H3mm18 NCP may be different from those of the H3.3 NCP.

**Figure 2.**
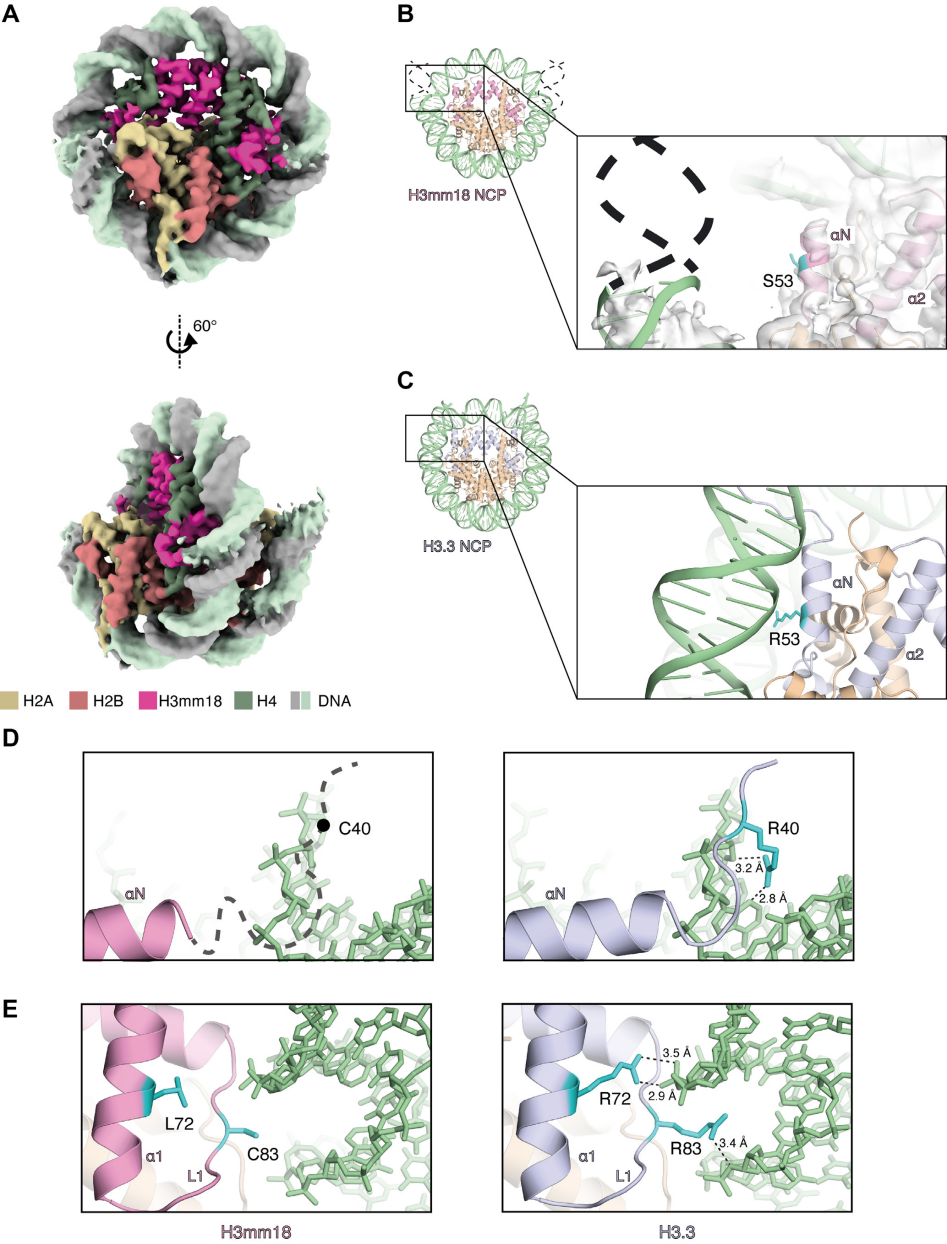
Cryo-EM structure of the NCP containing H3mm18. (**A**) The cryo-EM density map of the H3mm18 NCP. Histones H3mm18, H2A, H2B and H4 are colored pink, yellow, salmon pink, and green, respectively. DNA is colored gray and light green. (**B**) An entry/exit DNA region of the H3mm18 NCP. A close-up view is shown in the right panel. The H3mm18 Ser53 residue is colored cyan. The cryo-EM map is colored gray. The dashed lines show the putative DNA region that is disordered in the H3mm18 NCP. (**C**) An entry/exit DNA region of the H3.3 NCP (PDB ID: 5XM0). A close-up view is shown in the right panel. The H3 Arg53 residue is colored cyan. (**D**) Comparison of the entry/exit regions between the H3mm18 NCP (left panel) and H3.3 NCP (right panel). In the left panel, the thick dashed line with the putative Cys40 position shows the N-terminal disordered region of H3mm18 in the NCP. (**E**) Structural comparison around positions 72 and 83 in the H3mm18 NCP (left panel) and H3.3 NCP (right panel). In the left panel, the H3mm18 Leu72 and Cys83 residues are shown in cyan with side chains. In the right panel, the H3 Arg72 and Arg83 residues are shown in cyan with side chains. Possible hydrogen bonds are shown with dashed lines.

We obtained the H3mm18 NCP structure with a crosslinked sample, prepared by the GraFix method, at 3.6 Å resolution (Figure 2A, Supplementary Figure S2, and Table 1). We also prepared the H3mm18 NCP sample for the cryo-EM analysis in the presence of the PL2-6 single-chain antibody variable fragment (scFv), which stabilizes the cryo-EM nucleosome sample by binding to the acidic patch of the NCP (32). We determined the H3mm18 NCP structure at 3.4 Å resolution without crosslinking (Supplementary Figures S3, S4, and Table 1). The overall H3mm18 NCP structures are similar between the crosslinked and non-crosslinked samples. In both H3mm18 NCP structures, the entry/exit regions of the nucleosomal DNA are drastically disordered, and the central 125−130 bp region of the DNA is visibly wrapped around the histone octamer (Figures 2A, B, and Supplementary Figure S3). In contrast, in the H3.3 NCP, the entry/exit DNA regions are captured by the αN helix of H3, and 145 bp of DNA are clearly visible (Figure 2C) (4,29,65–67).

Intriguingly, the EM density of the H3mm18 αN helix was ambiguous (Figure 2B), suggesting that the αN helix region is unstable in the H3mm18 NCP. In the H3.3 NCP, the H3 Arg53 residue in the αN helix is suggested to play an important role in maintaining the histone-DNA contacts at the entry/exit DNA regions (68) (Figure 2C). Since the H3 Arg53 residue is replaced by a Ser residue in H3mm18, the histone-DNA contact at the H3 position 53 may be abolished in the H3mm18 NCP (Figure 2B). In the H3.3 NCP, the H3 Arg40, Arg72 and Arg83 residues directly bind to the DNA backbone (Figure 2D and E, right panels). In H3mm18, the neutral Cys40, Leu72 and Cys83 residues replace these basic Arg residues, and may reduce the histone-DNA contacts at these sites (Figure 2D and E, left panels). These histone−DNA interactions missing in the H3mm18 NCP may reduce its stability.

### The H3mm18 NCP is extremely unstable

**Figure 3.**
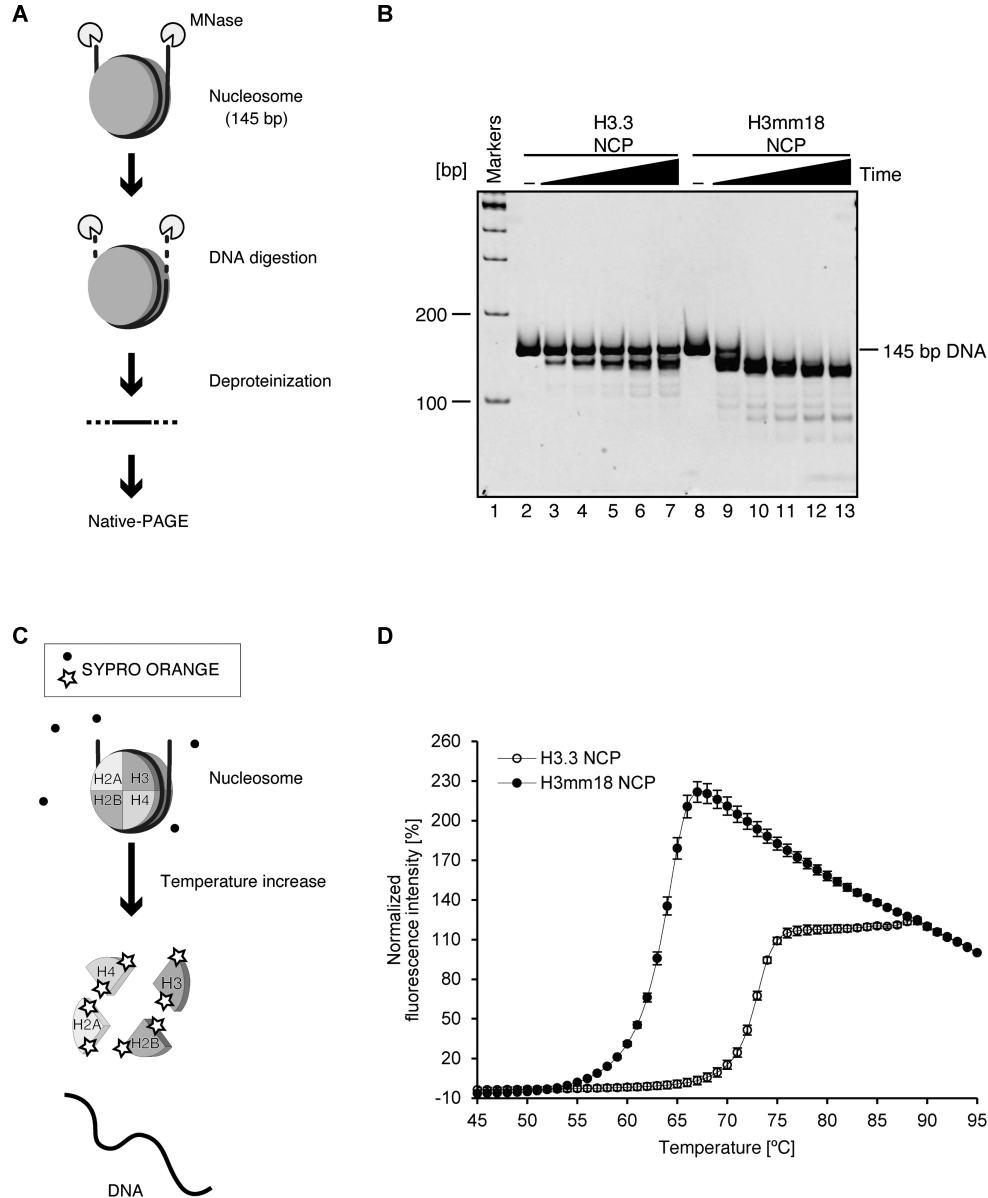
DNA end flexibility and instability of the H3mm18 NCP. (**A**) Schematic representation of the micrococcal nuclease (MNase) treatment assay. In this assay, the NCP is incubated in the presence of MNase, which preferentially digests the DNA regions detached from the histone proteins. After deproteinization, the resulting DNA fragment is analyzed by native-PAGE. (–) A representative gel image of the MNase treatment assay. The H3.3 (lanes 2-7) and H3mm18 (lanes 8-13) NCPs were incubated in the presence of MNase for 0, 3, 6, 9, 12 and 15 min. The resulting DNA fragments were analyzed by native-PAGE with ethidium bromide staining. The results were confirmed by two additional, independent experiments (Supplementary Figure S5). (**C**) Schematic representation of the thermal stability assay. In this assay, the histone proteins dissociated from the NCP by thermal denaturation are detected by the SYPRO Orange fluorescent dye, which binds the hydrophobic surface of the histones. (**D**) Normalized fluorescence intensity curves of thermal disruption of the H3.3 and H3mm18 NCPs. The error bars indicate the S.D. (*n* = 3).

The cryo-EM structure of the H3mm18 NCP revealed unusual DNA flexibility at the entry/exit regions. To test whether this characteristic is actually maintained in solution, we performed a micrococcal nuclease (MNase) assay. Since MNase preferentially digests the DNA end regions detached from the histone surface in the NCP, the DNA region tightly bound to the histone surface is generally protected from MNase (Figure 3A). As shown in Figure 3B, the DNA ends of the H3mm18 NCP were quite susceptible to MNase, as compared to those of the H3.3 NCP. These results are perfectly consistent with the cryo-EM structure of the H3mm18 NCP, in which the entry/exit DNA regions are flexibly disordered and exposed to the solvent.

In the H3mm18 NCP structure, we found fewer histone–DNA interactions, which may cause the instability of the H3mm18 NCP. We then tested the thermal stability of the H3mm18 NCP. In this assay, the histones released from the NCP by thermal denaturation are detected by binding SYPRO Orange fluorescent dye (Figure 3C). As shown in Figure 3D, the H3mm18 NCP was drastically unstable as compared with the H3.3 NCP. In fact, the H3mm18 NCP became denatured at a temperature about 10°C lower than that of the H3.3 NCP (Figure 3D). Therefore, we concluded that the H3mm18 NCP is extremely unstable, probably by the reduced histone-DNA interactions, as observed in its cryo-EM structure (Figure 2B, D and E, left panels).

### Contributions of the H3mm18 residues to the DNA flexibility and instability of the H3mm18 NCP

**Figure 4.**
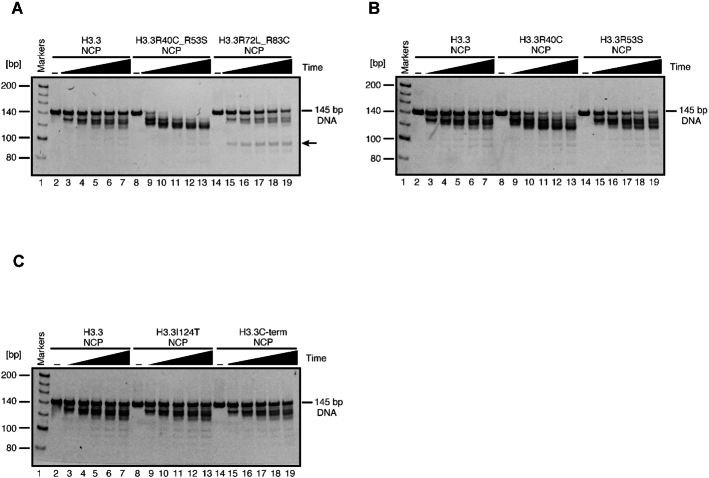
MNase treatment assay of the NCPs containing the H3.3 mutants. (**A**) The NCPs containing H3.3 (lanes 2–7), H3.3R40C_R53S (lanes 8–13) and H3.3R72L_R83C (lanes 14–19) were incubated in the presence of MNase for 0, 3, 6, 9, 12 and 15 min. (**B**) The experiments with the NCPs containing H3.3 (lanes 2–7), H3.3R40C (lanes 8–13) and H3.3R53S (lanes 14–19). (**C**) The experiments with the NCPs containing H3.3 (lanes 2–7), H3.3I124T (lanes 8–13) and H3.3C-term (lanes 14–19). The resulting DNA fragments were deproteinized and analyzed by native-PAGE with ethidium bromide staining. The results were confirmed by three additional independent experiments. The arrow in panel (A) indicates the band (90-95 base pairs) corresponding to the DNA fragment produced by MNase cleavage around SHL2.5.

We next tested whether the H3mm18-specific histone–DNA interactions are responsible for the DNA end flexibility and thermal stability of the H3mm18 NCP. To do so, we prepared the NCPs containing the H3.3 mutants, in which the Arg40, Arg53, Arg72 and Arg83 residues were replaced by the corresponding H3mm18 residues (H3.3R40C, H3.3R53S, H3.3R40C_R53S and H3.3R72L_R83C) (Supplementary Figure S6). The NCP containing the H3.3 C-terminal mutant (H3.3C-term), in which the H3.3 Arg128, Arg129, Arg131, Gly132 and Arg134 residues were replaced by the corresponding H3mm18 Gly128, Tyr129, Cys131, Arg132 and Cys134 residues, respectively, was also prepared (Supplementary Figure S6). In addition, the NCP containing the H3.3I124T mutant, in which the H3.3 Ile124 residue was replaced by the corresponding H3mm18 Thr residue, was prepared (Supplementary Figure S6). The MNase assay revealed that the H3.3 Arg40 and Arg53 double replacements clearly enhanced the DNA end susceptibility (Figure 4A). The H3.3 Arg40 or Arg53 single replacement also enhanced the DNA end susceptibility, although the effect of each single replacement was somewhat weak as compared to the double replacements (Figure 4B). These results suggested that the replacement of the Arg40 and Arg53 residues to neutral Cys and Ser in H3mm18, respectively, may be responsible for the DNA end flexibility in the H3mm18 NCP. On the other hand, the H3.3C-term mutations and the H3.3I124T mutation did not substantially affect the MNase susceptibility at the DNA ends (Figure 4C). However, interestingly, the NCP containing the H3.3R72L_R83C mutation revealed the presence of a new cleavage site, thus producing an ∼90–95 bp DNA fragment (Figure 4A). This may be caused by the enhanced MNase susceptibility around positions 72 and 83 (superhelical location (SHL) 2.5) of H3.3 in the NCP, suggesting that the histone-DNA interaction around the SHL2.5 may be weakened by the basic to neutral substitutions at these positions (Figure 2E).

**Figure 5.**
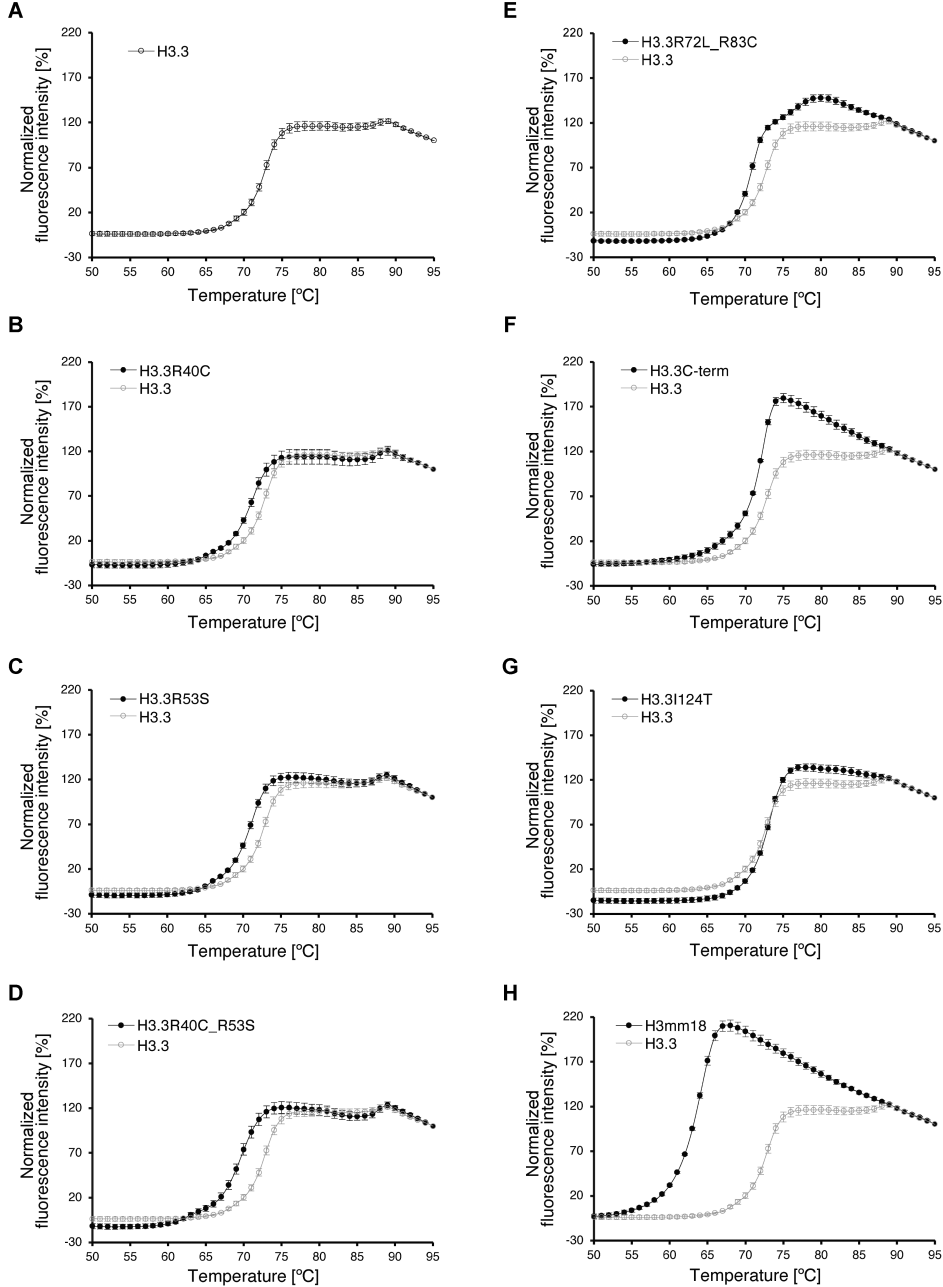
Thermal stability assay of the NCPs containing the H3.3 mutants. (**A**) Normalized fluorescence intensity curve of thermal disruption of the H3.3 NCP, presented with open circles. The error bars indicate the S.D. (*n* = 4). (B–G) Normalized fluorescence intensity curves of thermal disruption of the NCPs containing H3.3R40C (**B**), H3.3R53S (**C**), H3.3R40C_R53S (**D**), H3.3R72L_R83C (**E**), H3.3C-term (**F**), H3.3I124T (**G**) and H3mm18 (**H**), presented with closed circles. The error bars indicate the S.D. (*n* = 4). For comparison, the results with the H3.3 NCP shown in panel (A) are presented in gray. The error bars indicate the S.D. (*n* = 4).

The substitutions of the H3.3 Arg40, Arg53, Arg72 and Arg83 residues and the H3.3C-term mutations reduced the thermal stability of the H3.3 NCP (Figure 5A–F). However, the NCPs containing these amino acid substitutions were still more stable than the H3mm18 NCP (Figure 5B–F and H). Therefore, these H3.3 Arg residues directly bound to the nucleosomal DNA contribute to the H3.3 NCP stability, but each contribution is apparently partial. It should be noted that the thermal stability of the NCP containing the H3.3I124T substitution was not significantly affected (Figure 5G). The H3mm18 Thr124 residue is located in the hydrophobic cluster formed with H4 (Supplementary Figure S7), but may not substantially weaken the hydrophobic interaction in the NCP.

### Forced expression of H3mm18 suppresses myogenic differentiation

**Figure 6.**
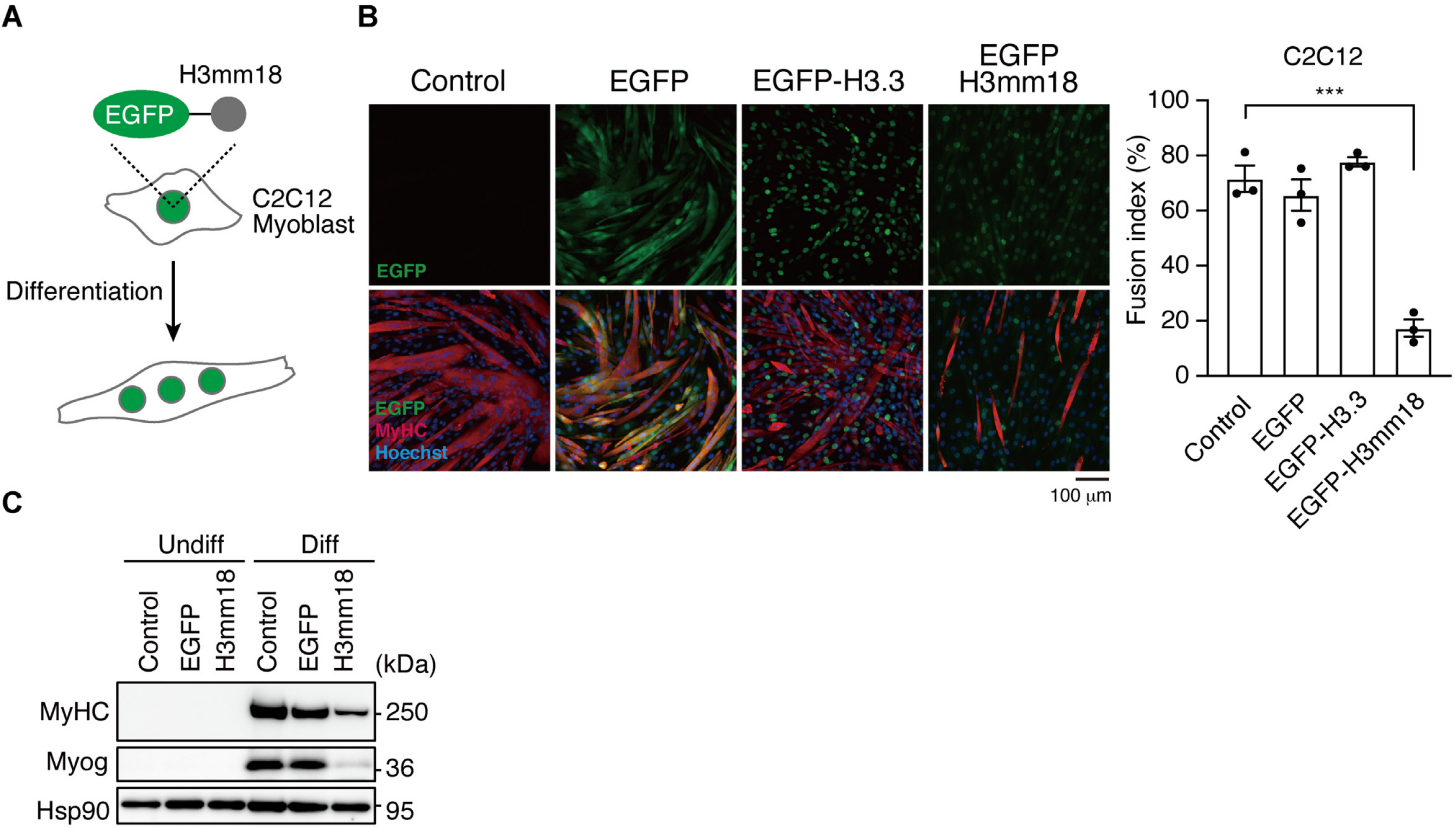
Ectopic expression of H3mm18 suppresses myogenic differentiation in C2C12 cells. (**A**) Schematic illustration of the ectopic expression experiment. (**B**) ICC analysis of differentiated C2C12 cells. C2C12 cells expressing EGFP, EGFP-tagged H3.3 or H3mm18 (green in bottom images, N-terminally tagged) were differentiated for 72 h and stained with antibodies for the indicated proteins. To evaluate the fusion index, the total nuclei and those in MyHC-positive myotubes were counted for each field of view (FOV, >300 nuclei for each FOV, three FOVs for each cell type). Fusion index is graphed as % MyHC-containing nuclei. ***P < 0.001; one-way analysis of variance (ANOVA). (**C**) Immunoblot analysis of undifferentiated (Undiff) and differentiated (Diff) C2C12 cells expressing EGFP or EGFP-H3mm18.

We next tested whether the unusual H3mm18 nucleosome formation affects the cellular function. H3mm18 is weakly but specifically expressed in skeletal muscle (27), suggesting its function in skeletal muscle differentiation. In previous studies, we have analyzed the physiological functions of the forced expression of specific histones (27,69,70). We applied this approach to evaluate the skeletal muscle differentiation potential through morphological changes and a transcriptome analysis, using skeletal myoblast C2C12 cells stably expressing H3mm18 (Figure 6A).

We also evaluated the physiological impact of H3mm18 on skeletal muscle differentiation. We quantified the H3mm18 expression level using the published RNA-seq data, which revealed that *H3mm18* was dominantly expressed in quiescent satellite cells and downregulated upon differentiation (Supplementary Figure S8A) (71). Since H3mm18 expression was not detected in C2C12 cells (Supplementary Figure S8B), we established C2C12 cells that stably expressed EGFP-tagged H3mm18 (EGFP-H3mm18) or H3.3 (EGFP-H3.3), following the deletion of the endogenous *H3mm18* gene locus by CRISPR/Cas9 (Supplementary Figure S8C and D), to avoid unfavorable endogenous H3mm18 expression. The *H3mm18* KO cells showed myogenic and housekeeping gene expressions at the same levels of WT cells (Supplementary Figure S8E–G). Interestingly, an immunocytochemistry (ICC) analysis demonstrated that the number of cells positive for MyHC, a marker of skeletal muscle differentiation, was obviously reduced among the EGFP–H3mm18-expressing cells at 72 h of differentiation. A fusion index analysis indicated a significant reduction (*P* < 0.001) in multinucleated myofiber formations in EGFP–H3mm18-expressing cells (20%), whereas EGFP- or EGFP–H3.3-expressing cells showed similar levels to the controls (60–80%) (Figure 6B). Furthermore, the ectopic expression of H3mm18 repressed MyHC and Myog, markers of skeletal muscle differentiation, to protein levels below those of the differentiated condition (Figure 6C). These results suggested that the ectopic expression of H3mm18 suppresses the differentiation of C2C12 cells.

### Forced expression of H3mm18 alters transcriptome in C2C12 cells

Our previous analyses revealed that the forced expression of H3.3 and its subvariant H3mm7 enhances gene expression genome-wide and activates skeletal muscle differentiation through chromatin incorporation (29). In contrast, the forced expression of H3mm18 suppressed skeletal muscle differentiation in C2C12 cells. To explore the mechanism of this H3mm18-induced suppression of skeletal muscle differentiation, we performed a transcriptome analysis using CEL-Seq2 to identify the genes affected by H3mm18.

**Figure 7.**
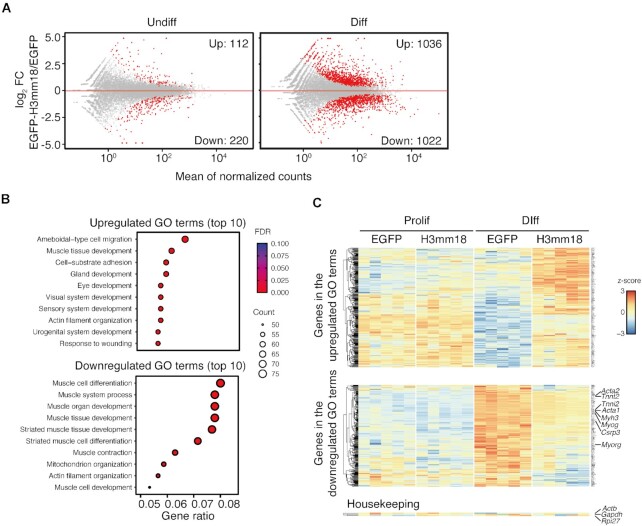
Ectopic expression of H3mm18 alters the transcription status in C2C12 cells. (**A**) Transcriptome analysis using CEL-Seq2. MA plots showing the effects of differential expressions of EGFP-H3mm18 on proliferative and differentiated states. Differentially expressed genes (DEGs) are depicted in red. (**B**) Gene ontology (GO) analysis on DEGs in differentiated cells. Top 10 GO terms for upregulated DEGs (left) and down-regulated DEGs (right panel) are listed. (**C**) A heatmap showing expression patterns of up- or down-regulated DEGs upon H3mm18 expression during differentiation. *Actb*, *Gapdh*, and *Rpl27* are shown as non-DEG, housekeeping genes.

We detected differentially expressed genes (DEGs) that were altered by the forced expression of H3mm18 in C2C12 cells before and after differentiation. The results showed that many DEGs were detected in post-differentiated cells (up: 1092, down: 1106) (Figure 7A). Similar numbers of DEGs were detected in both suppressed and enhanced expression. These results suggested that H3mm18 may act on genes that are induced and repressed during differentiation. We then used gene ontology (GO) to analyze the characteristics of the 2198 DEGs after the induction of differentiation. The data showed that the inhibitory group contains many terms that are highly relevant to muscle tissue, including muscle cell differentiation, muscle system process, and muscle tissue development (Figure 7B). These results indicated a tendency for suppressed gene expression during skeletal muscle differentiation. Conversely, the DEGs showing enhanced gene expression included a wide range of categories, and no specific categories were enriched. Consistently, we confirmed that certain individual genes, which are representatively upregulated among terminal differentiation markers of differentiation, were markedly suppressed: *Acta1* (log_2_FC: −2.07, FDR: 0.000039), *Tnnt2* (log_2_FC: −2.20, FDR: 0.00028), *Tnni2* (log_2_FC: −2.24, FDR: 0.0007), and *Myh3* (log_2_FC: −1.98, FDR: 0.049) (Figures 7C and Supplementary Figure S9). Furthermore, the expression of *Myog* (log_2_FC: −1.70, FDR: 0.0000018), a downstream factor of MyoD that regulates these genes, was also repressed. This indicated that the genes related to skeletal muscle differentiation are extensively repressed at the upstream regulatory level, probably through the altered chromatin conformation by the incorporation of the forced expressed H3mm18, whereas no significant effect was observed for genes that are ubiquitously expressed.

### H3mm18 affects myogenic lineage gene expression

**Figure 8.**
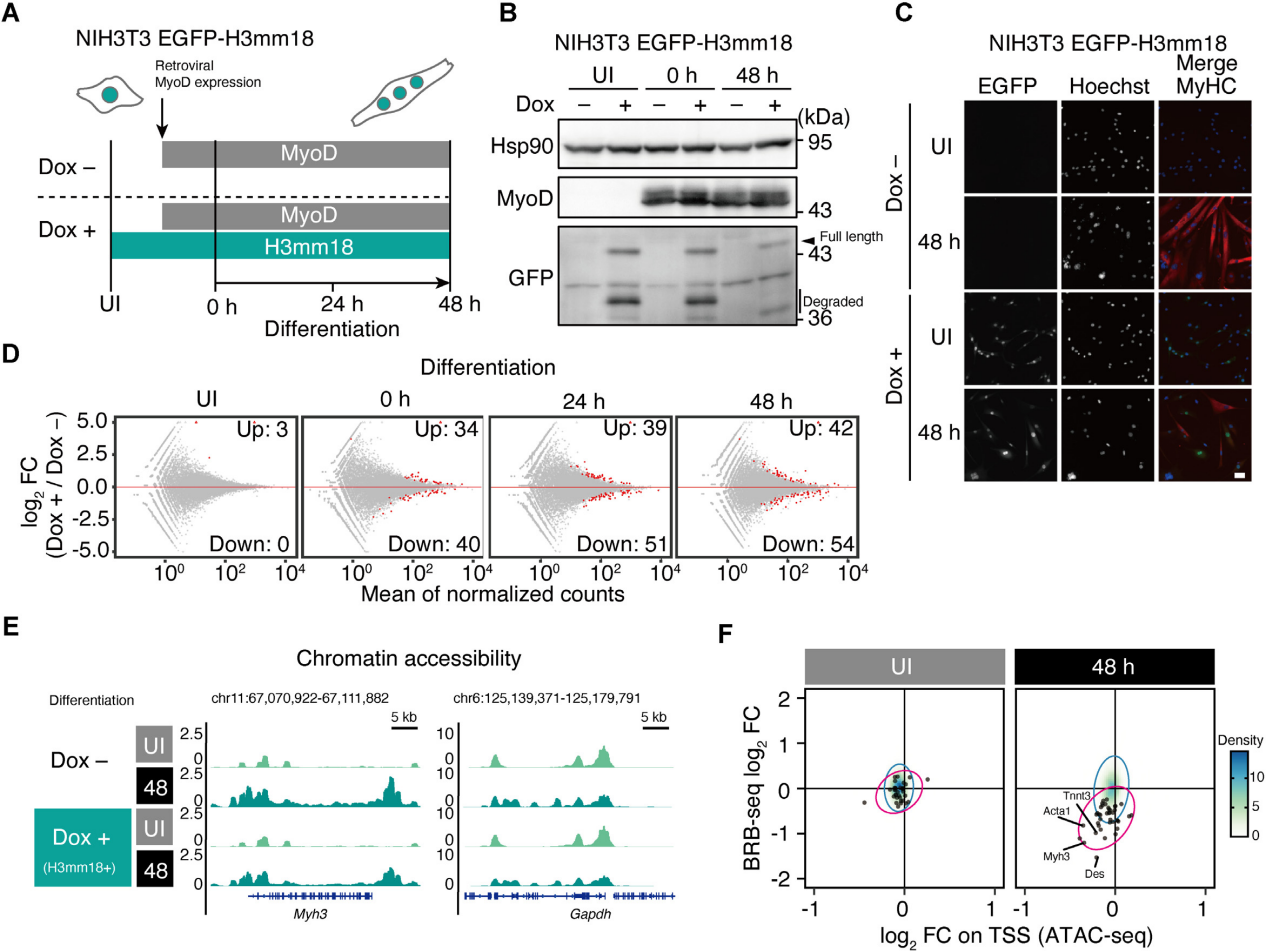
H3mm18 expression alters chromatin structures on myogenic gene loci during differentiation. (**A**) Schematic illustration of trans-differentiation experiments. NIH3T3 cells, expressing a doxycycline (Dox)-inducible EGFP-H3mm18 construct, were cultured with or without Dox for 24 h and subjected to retroviral infection-expressing MyoD. Differentiation was induced after 30 h of the retroviral infection. UI (UnInfected) indicates the time before retroviral infection, and 0, 24 and 48 h indicate the times after differentiation induction. (**B**) Immunoblot analysis of NIH3T3 EGFP-H3mm18 cells. (**C**) Immunocytochemistry (ICC) analysis of NIH3T3 EGFP-H3mm18 cells ectopically expressing MyoD. Cells at UI and 48 h were stained with an anti-MyHC antibody. DNA was counterstained with Hoechst 33342. Scale bar: 50 μm. (**D**) MA plots showing the time-series transcriptome profiles of the trans-differentiation experiments. (**E**) Representative genome browser images of ATAC-Seq at the *Myh3* gene locus. (**F**) Time-series correlation analysis of the effects of transcriptome and open-chromatin profile alterations on H3mm18 expression. Fold changes of gene expression and ATAC peak signals on the TSS are plotted. Black dots indicate the 54 downregulated DEGs at 48 hours of differentiation. Red and blue ellipses indicate the 95% confidence intervals of the 54 DEGs in (D) and total genes, respectively.

To determine whether the native functions of H3mm18 are affected, we used a MyoD-induced skeletal muscle differentiation system with NIH3T3 cells for discriminating the myogenic dependency of H3mm18 function (29,69). We established NIH3T3 cells that express N-terminally EGFP-tagged H3mm18 (EGFP–H3mm18) dependent on doxycycline (Dox) (Figure 8A and B). Next, we performed a set of RNAseq for UI (uninfected), 0, 24 and 48 h after differentiation upon MyoD induction with or without Dox. The transcriptome changes upon H3mm18 production were observed at 0, 24 and 48 h of differentiation, but not for UI, which did not have MyoD induction (Figure 8D). The ICC analysis showed that the number of MyHC-positive cells, a marker of late myogenesis, was reduced by the Dox treatment at 48 h of differentiation (Figure 8C). These results suggested that the effect of H3mm18 is dependent on MyoD-driven gene expression upon skeletal muscle differentiation.

We performed further ATAC-seq analyses to evaluate the chromatin accessibility of MyoD-induced NIH3T3 cells, at UI and 48 h after differentiation. We will describe a representative ATAC-seq result on the *Myh3* gene locus. H3mm18 expression caused a decrease in the chromatin accessibility around the *Myh3* gene at 48 h, while no significant change was observed at UI (Figure 8E). The decreased chromatin accessibility was also found in 54 genes downregulated by H3mm18 expression at 48 h (Figure 8F). Decreased chromatin accessibility is generally considered to correlate with gene suppression. Therefore, these results are consistent with the idea that forced H3mm18 expression downregulated a class of genes through the decreased chromatin accessibility. The H3mm18 incorporated in chromatin may induce the formation of an inaccessible chromatin architecture by the specific structural properties of its NCP, or by the rapid exchange of the incorporated H3mm18 to H3.1 or H3.2, although the mechanism has not been clarified yet.

### H3mm18 interacts with the H3.3 chaperone

**Figure 9.**
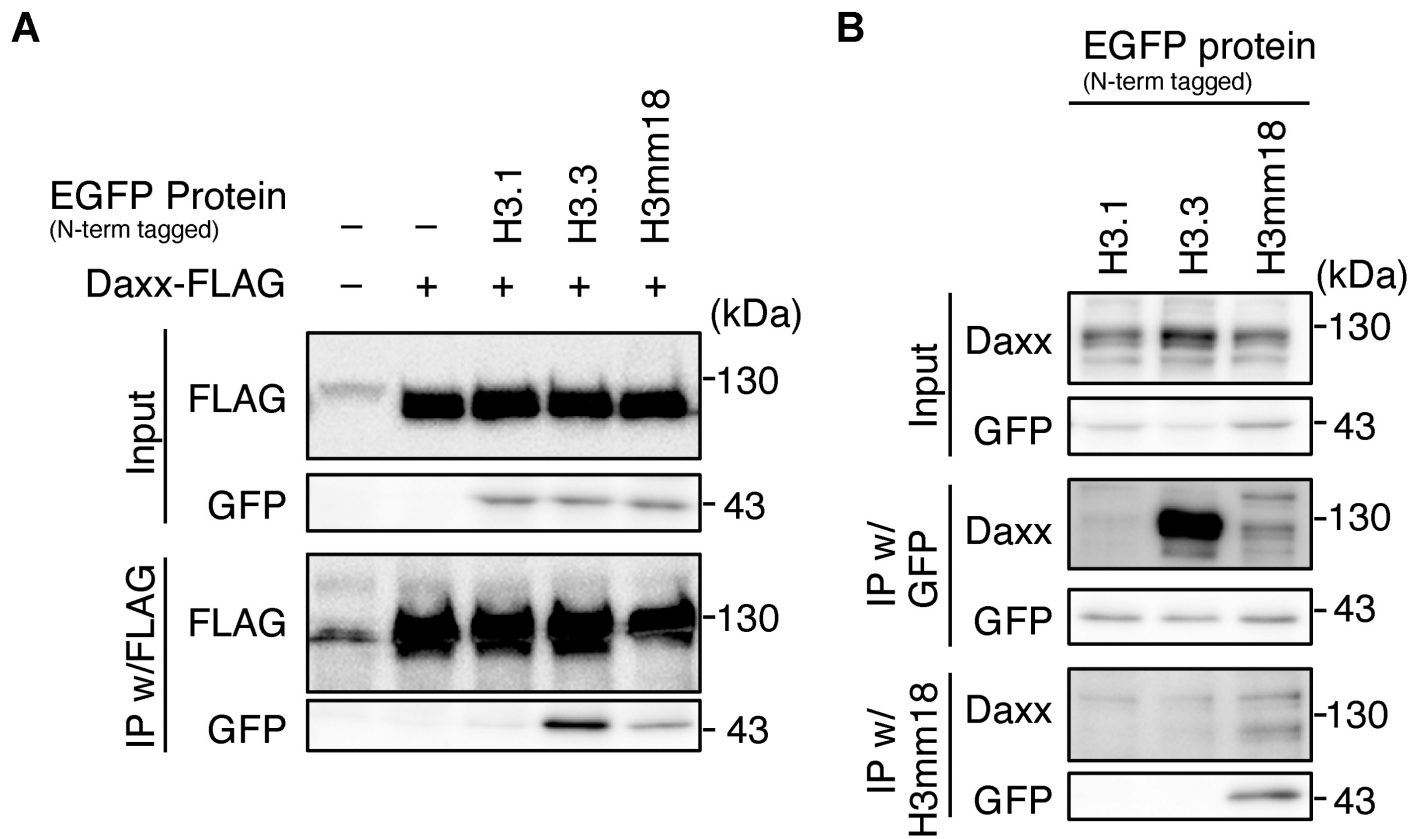
H3mm18 interacts with H3.3 chaperones. (**A**) Coimmunoprecipitation with FLAG-tagged DAXX. NIH3T3 cells expressing the N-terminally EGFP-tagged histone H3.1, H3.3 or H3mm18 were infected with retroviral vectors for C-terminally FLAG-tagged DAXX, and then treated with 5 μM MG132 for 6 h. Chromatin-unbound fractions were prepared and subjected to the immunoprecipitation (IP) assay using the anti-FLAG antibody. Input fractions and precipitates were analyzed by western blotting using the indicated antibodies. (**B**) IP for interaction with endogenous DAXX. NIH3T3 cells expressing the N-terminally EGFP-tagged histone H3.1, H3.3 or H3mm18 were treated with 5 μM MG132 for 6 h and subjected to IP, using anti-GFP or anti-H3mm18 antibodies.

Since H3.3 and H3mm18 share the chaperone-recognition domain of H3.3 (the Ala–Ala–Ile–Gly motif) (18,27) (Figure 1A), we examined the association of H3mm18 to the H3.3 chaperone. A representative H3.3 chaperone, DAXX, was tagged with FLAG and expressed in the NIH 3T3 cells. Co-immunoprecipitation assays were performed with EGFP-tagged H3.1, H3.3 or H3mm18. H3mm18 is a highly unstable protein, and therefore a proteasome inhibitor (MG-132) was used to stabilize the protein, as in our previous study (27). The data showed that the FLAG-tagged DAXX co-immunoprecipitated with the H3mm18 protein but not with EGFP-tagged H3.1, which has a different chaperone recognition motif (Figure 9A). Reciprocal experiments were also performed to confirm the specific binding of H3mm18 to DAXX, as well as to H3.3 (Figure 9B). H3mm18 was detected with the original monoclonal antibody, which was validated as shown in Supplementary Figure S10A and B. These results indicated that H3mm18 can be recognized by an H3.3 chaperone.

## DISCUSSION

H3mm18 is one of the *Mus musculus* H3 variants identified by *in silico* hybridization screening (27). However, its structural and biochemical characteristics have remained enigmatic. In the present study, we first found that H3mm18 was incorporated into chromatin in cells, although with very low efficiency as compared to H3.3 (Figure 1B, C). Consistently, the H3mm18 NCP was substantially unstable *in vitro* (Figure 3D). The NCP instability found in the H3mm18 NCP may play a role in the regulation of genomic DNA function in chromatin. Actually, our results demonstrated that the forced expression of H3mm18 drastically suppressed the differentiation of C2C12 myoblasts into myotubes, by causing distinctive changes in the gene expression profile (Figures 6 and 7). These results suggested that H3mm18 may function to regulate the appropriate gene expression profile for muscle differentiation by altering the chromatin conformation with the unusual H3mm18 NCP formation.

Among H3 variants, the human histone H3T and H3.5 variants, which are produced in testicular cells, form unstable NCPs (72,73). Previous studies revealed that the H3T Val111 and H3.5 Leu103 residues are responsible for the NCP instability (72,73). These residues are located in the four-helix bundle formed between H3 and H3 in the nucleosome. Amino acid substitutions in or near the four-helix bundle region, such as *Sin2* mutations, destabilize the nucleosome (74,75). The *Sin2* mutations can relieve the requirement of a nucleosome remodeler for transcription (74), suggesting that the NCP instability induced by the histone mutations may reduce the nucleosome barrier during transcription (5,9–10,76,77). In the four-helix bundle region, an amino acid substitution at position 124 is found in the human H3.Y variant (78,79), which contains the hydrophobic Met124 residue. Interestingly, the H3.Y-specific Met124 residue plays a role in enhancing the stability of the NCP (80). The H3mm18-specific Thr124 residue is also present in this position. However, the substitution of the hydrophobic H3.3 Ile124 residue with the hydrophilic Thr124 residue did not significantly reduce the H3.3 NCP stability (Figure 5G). In the H3mm18 NCP structure, the H3mm18 Thr124 residue is involved in the hydrophobic cluster with H4, and the Ile to Thr substitution at position 124 may not substantially affect the structure of this hydrophobic cluster (Supplementary Figure S7). The Thr124 residue may somewhat weaken the hydrophobic histone-histone interactions in the four-helix bundle, but not to the point of inducing detectable NCP instability by the H3.3 I124T substitution. The weakened hydrophobic interaction with the Thr124 residue in the NCP may be magnified in combinations with the other amino acid substitutions of H3mm18.

The cryo-EM structures of the H3mm18 NCP revealed that H3mm18 induced drastic structural changes in the entry/exit DNA regions, when it was incorporated into the nucleosome (Figures 2 and Supplementary Figure S3). Previous studies on other histone variants suggested the importance of the flexible nucleosomal DNA ends in physiological functions. A representative example is the NCP containing the centromere-specific histone H3 variant, CENP-A. In the CENP-A NCP, the entry/exit DNA regions are flexible (81,82). Human CENP-A contains a Lys amino acid substitution at position 53, which corresponds to the Arg residue in the canonical H3. A molecular dynamics simulation study suggested that the CENP-A Lys53 residue may play a role in unwrapping the entry/exit DNA regions in the NCP (68). In H3mm18, the 53rd residue is replaced by Ser, which may weaken the histone-DNA contacts at the entry/exit DNA regions, and function in the DNA end flexibility, together with the Cys40 residue (Figure 4A). Asymmetric DNA detachment of the DNA ends has been reported in the cryo-EM analysis of the CENP-A NCP (83). Similarly, the asymmetric DNA detachment is also obvious in the H3mm18 NCPs (Figures 2A and Supplementary Figure S3). Intriguingly, the CENP-A mutant, which abolishes the entry/exit DNA flexibility in the NCP, exhibited serious defects in the centromere function in cells (84). This indicated that the entry/exit DNA flexibility of the CENP-A NCP actually functions to maintain active centromeres in cells. The DNA end flexibility of the CENP-A nucleosome suppresses the nucleosome binding of the linker histone H1, and the H1 elimination from the CENP-A nucleosome may play an important role in the establishment of functional centromeres (84). The DNA flexibility at the nucleosomal entry/exit regions has also been reported in the NCPs containing other histone variants, such as human H3.Y (78), mouse H3t (28), human H2A.B (85–87), human H2A.Z (88) and mouse H2A.L.2 (89,90). Since the entry/exit DNA flexibility affects the nucleosome arrangement in polynucleosomes (91), the linker histone-nucleosome binding (78,92), and nucleosome remodeling (93), the unusual entry/exit DNA flexibility and asymmetry found in the H3mm18 NCP may affect the high-order chromatin architecture around the genomic region with this histone variant.

Substantial DNA end flexibility has been reported in the NCP containing a histone H2A variant, H2A.B (formerly H2A.Bbd), which is incorporated into chromatin at replication and repair sites (86,94,95). H2A.B is efficiently exchanged in cells (95,96), probably through the formation of the open NCP conformation (87). The DNA end flexibility of the H2A.B NCP has been observed by cryo-EM (97). Deletions of the H2A C-terminal domain (docking domain) resulted in high flexibility of the nucleosomal DNA ends (83), indicating that the enhanced DNA-end flexibility of the H2A.B NCP may be responsible for the shortened C-terminal domain, which directly binds to the N-terminal region of H3. Interestingly, the N-terminal regions of H3mm18 were flexibly disordered in the H3mm18 NCP (Figures 2 and Supplementary Figure S3). This suggests that the interaction between the H3mm18 N-terminal domain and the H2A C-terminal domain may be weakened in the H3mm18 NCP, and thus may enhance the DNA end flexibility.

Our transcriptome analyses revealed that the forced expression of H3mm18 alters the transcription status of genes by both up- and down-regulation (Figure 7). In this situation, H3mm18 may be widely incorporated in the genome. The nucleosome is the major roadblock for transcription by RNA polymerase II (76,77). Considering the instability of the H3mm18 NCP, the up-regulation of genes by the forced H3mm18 expression may be explained by the alleviation of the nucleosome barrier during transcription, due to the incorporation of the unstable H3mm18 nucleosome in the coding and/or regulatory regions of up-regulated genes. However, under the forced H3mm18 expression conditions, we also found many genes, including myogenic genes, that were down-regulated (Figure 7). This may occur by three possible pathways. First, the unstable H3mm18 nucleosomes formed around the genes involved in muscle differentiation may be exchanged by the nucleosomes containing H3.1 and H3.2, which have a propensity to repress transcription (23). The H3mm18 nucleosome assembled around the down-regulated genes may also be replaced by histone H3 with suppressive post-translational modifications (93). Second, the unusual structure of the H3mm18 nucleosome may change the higher order chromatin conformation from an active to suppressive form in the transcribed regions of the genome. Third, H3mm18 may compete with H3.3 for binding to histone chaperones, such as DAXX (Figure 9), and thus indirectly inhibit the transcriptionally active chromatin containing H3.3. These possibilities are consistent with the observation that the chromatin accessibilities around the TSSs of downregulated myogenic DEGs were reduced by the stable expression of H3mm18 (Figure 8E and F). These aberrant chromatin compositions and conformations induced by H3mm18 may affect gene expression.

In the NIH3T3 trans-differentiation system, overexpression of H3mm18 did not affect the overall transcriptome of NIH3T3. However, the MyoD-induced transcriptome alteration was impaired by *H3mm18* overexpression, resulting in the suppression of differentiation. This is different from the effect of H3mm7, which is independent of myogenic gene expression upon ectopic MyoD expression (29). Although H3mm7 and H3mm18 both form NCPs, the H3mm18 NCP exhibited drastically unstable characteristics as compared to the H3mm7 NCP (Figures 3 and 5). The DNA end flexibility of the H3mm18 NCP may also be higher than that of the H3mm7 NCP (Figures 2–4). These differences between the H3mm18 and H3mm7 NCPs may explain why H3mm18 has very limited potential for chromatin incorporation, unlike H3mm7 (29). It is also possible that H3mm18 has another function besides serving as an architectural chromatin component. Further studies are awaited to clarify the mechanism by which H3mm18 regulates genes in chromatin.

## DATA AVAILABILITY

Atomic coordinates and cryo-EM maps have been deposited in the PDB and EMDB under accession numbers 7DBH and EMD-30631 (the crosslinked H3mm18 NCP), and 7VBM and EMD-31882 (the H3mm18 NCP aided by PL2-6 scFv). The RNA-Seq data in this study were deposited in the Gene Expression Omnibus (GEO) under the following accession code: GSE: 168238. The codes used for our sequencing data analysis are available at https://github.com/AMyKuwa/18project_2020.

## Supplementary Material

gkab1137_Supplemental_File

## References

[1] Wolffe A.P. Chromatin: Structure and Function. 1998; 3rd edn San Diego Academic Press.

[2] Arents G., Burlingame R.W., Wang B.C., Love W.E., Moudrianakis E.N. The nucleosomal core histone octamer at 3.1 Å resolution: a tripartite protein assembly and a left-handed superhelix. Proc. Natl. Acad. Sci. U.S.A. 1991; 88:10148–10152. 1946434 10.1073/pnas.88.22.10148 PMC52885

[3] Luger K., Mäder A.W., Richmond R.K., Sargent D.F., Richmond T.J. Crystal structure of the nucleosome core particle at 2.8 Å resolution. Nature. 1997; 389:251–260. 9305837 10.1038/38444

[4] Luger K., Dechassa M., Tremethick D. New insights into nucleosome and chromatin structure: an ordered state or a disordered affair?. Nat. Rev. Mol. Cell Biol. 2012; 13:436–447. 22722606 10.1038/nrm3382 PMC3408961

[5] Petesch S.J., Lis J.T. Overcoming the nucleosome barrier during transcript elongation. Trends Genet. 2012; 28:285–294. 22465610 10.1016/j.tig.2012.02.005 PMC3466053

[6] Soria G., Polo S.E., Almouzni G. Prime, repair, restore: the active role of chromatin in the DNA damage response. Mol. Cell. 2012; 46:722–734. 22749398 10.1016/j.molcel.2012.06.002

[7] Bai L., Morozov A.V. Gene regulation by nucleosome positioning. Trends Genet. 2010; 26:476–483. 20832136 10.1016/j.tig.2010.08.003

[8] Venkatesh S., Workman J.L. Histone exchange, chromatin structure and the regulation of transcription. Nat. Rev. Mol. Cell Biol. 2015; 16:178–189. 25650798 10.1038/nrm3941

[9] Kobayashi W., Kurumizaka H. Structural transition of the nucleosome during chromatin remodeling and transcription. Curr. Opin. Struct. Biol. 2019; 59:107–114. 31473439 10.1016/j.sbi.2019.07.011

[10] Kujirai T., Kurumizaka H. Transcription through the nucleosome. Curr. Opin. Struct. Biol. 2019; 61:42–49. 31790919 10.1016/j.sbi.2019.10.007

[11] Koyama M., Kurumizaka H. Structural diversity of the nucleosome. J. Biochem. 2018; 163:85–95. 29161414 10.1093/jb/mvx081

[12] Talbert P.B., Meers M.P., Henikoff S. Old cogs, new tricks: the evolution of gene expression in a chromatin context. Nat. Rev. Genet. 2019; 20:283–297. 30886348 10.1038/s41576-019-0105-7

[13] Brahma S., Henikoff S. Epigenome regulation by dynamic nucleosome unwrapping. Trends Biochem. Sci. 2020; 45:13–26. 31630896 10.1016/j.tibs.2019.09.003 PMC10168609

[14] Malik H.S., Henikoff S. Phylogenomics of the nucleosome. Nat. Struct. Biol. 2003; 10:882–891. 14583738 10.1038/nsb996

[15] Bönisch C., Hake S.B. Histone H2A variants in nucleosomes and chromatin: more or less stable?. Nucleic Acids Res. 2012; 40:10719–10741. 23002134 10.1093/nar/gks865 PMC3510494

[16] Talbert P.B., Henikoff S. Histone variants on the move: substrates for chromatin dynamics. Nat. Rev. Mol. Cell Biol. 2017; 18:115–126. 27924075 10.1038/nrm.2016.148

[17] Buschbeck M., Hake S.B. Variants of core histones and their roles in cell fate decisions, development and cancer. Nat. Rev. Mol. Cell Biol. 2017; 18:299–314. 28144029 10.1038/nrm.2016.166

[18] Martire S., Banaszynski L.A. The roles of histone variants in fine-tuning chromatin organization and function. Nat. Rev. Mol. Cell Biol. 2020; 21:522–541. 32665685 10.1038/s41580-020-0262-8 PMC8245300

[19] Kurumizaka H., Kujirai T., Takizawa Y. Contributions of histone variants in nucleosome structure and function. J. Mol. Biol. 2020; 433:166678. 33065110 10.1016/j.jmb.2020.10.012

[20] Kaufman P.D., Kobayashi R., Kessler N., Stillman B. The p150 and p60 subunits of chromatin assembly factor I: a molecular link between newly synthesized histones and DNA replication. Cell. 1995; 81:1105–1114. 7600578 10.1016/s0092-8674(05)80015-7

[21] Marzluff W.F., Wagner E.J., Duronio R.J. Metabolism and regulation of canonical histone mRNAs: life without a poly(A) tail. Nat. Rev. Genet. 2008; 9:843–854. 18927579 10.1038/nrg2438 PMC2715827

[22] Tagami H., Ray-Gallet D., Almouzni G., Nakatani Y. Histone H3.1 and H3.3 complexes mediate nucleosome assembly pathways dependent or independent of DNA synthesis. Cell. 2004; 116:51–61. 14718166 10.1016/s0092-8674(03)01064-x

[23] Hake S.B., Allis C.D. Histone H3 variants and their potential role in indexing mammalian genomes: “H3 barcode hypothsis”. Proc. Natl. Acad. Sci. U.S.A. 2006; 103:6428–6435. 16571659 10.1073/pnas.0600803103 PMC1564199

[24] Loyola A., Almouzni G. Marking histone H3 variants: how, when and why?. Trends Biochem. Sci. 2007; 32:425–433. 17764953 10.1016/j.tibs.2007.08.004

[25] Brush D., Dodgson J.B., Choi O.R., Stevens P.W., Engel J.D. Replacement variant histone genes contain intervening sequences. Mol. Cell Biol. 1985; 5:1307–1317. 2863747 10.1128/mcb.5.6.1307-1317.1985 PMC366859

[26] Ray-Gallet D., Quivy J.P., Scamps C., Martini E.M., Lipinski M., Almouzni G. HIRA is critical for a nucleosome assembly pathway independent of DNA synthesis. Mol. Cell. 2002; 9:1091–1100. 12049744 10.1016/s1097-2765(02)00526-9

[27] Maehara K., Harada A., Sato Y., Matsumoto M., Nakayama K.I., Kimura H., Ohkawa Y. Tissue-specific expression of histone H3 variants diversified after species separation. Epigenet. Chromatin. 2015; 8:35. 10.1186/s13072-015-0027-3 PMC4574566 26388943

[28] Ueda J., Harada A., Urahama T., Machida S., Maehara K., Hada M., Makino Y., Nogami J., Horikoshi N., Osakabe A. et al. Testis-specific histone variant H3t gene is essential for entry into spermatogenesis. Cell Rep. 2017; 18:593–600. 28099840 10.1016/j.celrep.2016.12.065

[29] Harada A., Maehara K., Ono Y., Taguchi H., Yoshioka K., Kitajima Y., Xie Y., Sato Y., Iwasaki T., Nogami J. et al. Histone H3.3 sub-variant H3mm7 is required for normal skeletal muscle regeneration. Nat. Commun. 2018; 9:1400. 29643389 10.1038/s41467-018-03845-1 PMC5895627

[30] Kujirai T., Arimura Y., Fujita R., Horikoshi N., Machida S., Kurumizaka H. Methods for preparing nucleosomes containing histone variants. Methods Mol. Biol. 2018; 1832:3–20. 30073519 10.1007/978-1-4939-8663-7_1

[31] Lowary P.T., Widom J. New DNA sequence rules for high affinity binding to histone octamer and sequence-directed nucleosome positioning. J. Mol. Biol. 1998; 276:19–42. 9514715 10.1006/jmbi.1997.1494

[32] Zhou B.R., Yadav K.N.S., Borgnia M., Hong J., Cao B., Olins A.L., Olins D.E., Bai Y., Zhang P. Atomic resolution cryo-EM structure of a native-like CENP-A nucleosome aided by an antibody fragment. Nat. Commun. 2019; 10:2301. 31127102 10.1038/s41467-019-10247-4 PMC6534667

[33] Zhou B.R., Bai Y. Preparation of scFv stabilized chromatosomes for single-particle cryo-EM structure determination. STAR Protoc. 2021; 2:100396. 33786462 10.1016/j.xpro.2021.100396 PMC7994535

[34] Kastner B., Fischer N., Goals M.M., Sander B., Dube P., Boehringer D., Hartmuth K., Deckert J., Hauer F., Wolf E. et al. GraFix: sample preparation for single-particle electron cryomicroscopy. Nat. Methods. 2007; 5:53–55. 18157137 10.1038/nmeth1139

[35] Mastronarde D.N. Automated electron microscope tomography using robust prediction of specimen movements. J. Struct. Biol. 2005; 152:36–51. 16182563 10.1016/j.jsb.2005.07.007

[36] Zheng S.Q., Palovcak E., Armache J.P., Verba K.A., Cheng Y., Agard D.A. MotionCor2: anisotropic correction of beam-induced motion for improved cryo-electron microscopy. Nat. Methods. 2017; 14:331–332. 28250466 10.1038/nmeth.4193 PMC5494038

[37] Rohou A., Grigorieff N. CTFFIND4: Fast and accurate defocus estimation from electron micrographs. J. Struct. Biol. 2015; 192:216–21. 26278980 10.1016/j.jsb.2015.08.008 PMC6760662

[38] Zivanov J., Nakane T., Forsberg B.O., Kimanius D., Hagen W.J., Lindahl E., Scheres S.H. New tools for automated high-resolution cryo-EM structure determination in RELION-3. eLife. 2018; 7:e42166. 30412051 10.7554/eLife.42166 PMC6250425

[39] Vasudevan D., Chua E.Y.D., Davey C.A. Crystal structures of nucleosome core particles containing the ‘601’ strong positioning sequence. J. Mol. Biol. 2010; 403:1–10. 20800598 10.1016/j.jmb.2010.08.039

[40] Scheres S.H. Processing of structurally heterogeneous cryo-EM data in RELION. Methods Enzymol. 2016; 579:125–157. 27572726 10.1016/bs.mie.2016.04.012

[41] Kleywegt G.J., Harris M.R., Zou J.Y., Taylor T.C., Wählby A., Jones T.A. The uppsala electron-density server. Acta Crystallogr. D Biol. Crystallogr. 2004; 60:2240–2249. 15572777 10.1107/S0907444904013253

[42] Pettersen E.F., Goddard T.D., Huang C.C., Couch G.S., Greenblatt D.M., Meng E.C., Ferrin T.E. UCSF Chimera–a visualization system for exploratory research and analysis. J. Comput. Chem. 2004; 25:1605–1612. 15264254 10.1002/jcc.20084

[43] Kim D.N., Moriarty N.W., Kirmizialtin S., Afonine P.V., Poon B., Sobolev O.V., Adams P.D., Sanbonmatsu K. Cryo_fit: democratization of flexible fitting for cryo-EM. J. Struct. Biol. 2019; 208:1–6. 31279069 10.1016/j.jsb.2019.05.012 PMC7112765

[44] Liebschner D., Afonine P.V., Baker M.L., Bunkóczi G., Chen V.B., Croll T.I., Hintze B., Hung L.W., Jain S., McCoy A.J. et al. Macromolecular structure determination using X-rays, neutrons and electrons: recent developments in Phenix. Acta Crystallogr. D Struct. Biol. 2019; 75:861–877. 31588918 10.1107/S2059798319011471 PMC6778852

[45] Emsley P., Lohkamp B., Scott W.G., Cowtan K. Features and development of Coot. Acta Crystallogr. D Biol. Crystallogr. 2010; 66:486–501. 20383002 10.1107/S0907444910007493 PMC2852313

[46] Croll T.I. ISOLDE: a physically realistic environment for model building into low-resolution electron-density maps. Acta Crystallogr. D Struct. Biol. 2018; 74:519–530. 29872003 10.1107/S2059798318002425 PMC6096486

[47] Williams C.J., Headd J.J., Moriarty N.W., Prisant M.G., Videau L.L., Deis L.N., Verma V., Keedy D.A., Hintze B.J., Chen V.B. et al. MolProbity: more and better reference data for improved all-atom structure validation. Protein Sci. 2018; 27:293–315. 29067766 10.1002/pro.3330 PMC5734394

[48] Goddard T.D., Huang C.C., Meng E.C., Pettersen E.F., Couch G.S., Morris J.H., Ferrin T.E. UCSF ChimeraX: meeting modern challenges in visualization and analysis. Protein Sci. 2018; 27:14–25. 28710774 10.1002/pro.3235 PMC5734306

[49] Taguchi H., Horikoshi N., Arimura Y., Kurumizaka H. A method for evaluating nucleosome stability with a protein-binding fluorescent dye. Methods. 2014; 70:119–126. 25220913 10.1016/j.ymeth.2014.08.019

[50] Ishiguro K.I., Matsuura K., Tani N., Takeda N., Usuki S., Yamane M., Sugimoto M., Fujimura S., Hosokawa M., Chuma S. et al. MEIOSIN directs the switch from mitosis to meiosis in mammalian germ cells. Dev. Cell. 2020; 52:429–445. 32032549 10.1016/j.devcel.2020.01.010

[51] Hashimshony T., Senderovich N., Avital G., Klochendler A., de Leeuw Y., Anavy L., Gennert D., Li S., Livak K.J., Rozenblatt-Rosen O. et al. CEL-Seq2: sensitive highly-multiplexed single-cell RNA-Seq. Genome Biol. 2016; 17:77. 27121950 10.1186/s13059-016-0938-8 PMC4848782

[52] Smith T., Heger A., Sudbery I. UMI-tools: modeling sequencing errors in Unique Molecular Identifiers to improve quantification accuracy. Genome Res. 2017; 27:491–499. 28100584 10.1101/gr.209601.116 PMC5340976

[53] Kim D., Paggi J.M., Park C., Bennett C., Salzberg S.L. Graph-based genome alignment and genotyping with HISAT2 and HISAT-genotype. Nat. Biotechnol. 2019; 37:907–915. 31375807 10.1038/s41587-019-0201-4 PMC7605509

[54] Kim D., Langmead B., Salzberg S.L. HISAT: a fast spliced aligner with low memory requirements. Nat. Methods. 2015; 12:357–360. 25751142 10.1038/nmeth.3317 PMC4655817

[55] Pertea M., Kim D., Pertea G.M., Leek J.T., Salzberg S.L. Transcript-level expression analysis of RNA-seq experiments with HISAT, StringTie and Ballgown. Nat. Protoc. 2016; 11:1650–1667. 27560171 10.1038/nprot.2016.095 PMC5032908

[56] Liao Y., Smyth G.K., Shi W. featureCounts: an efficient general purpose program for assigning sequence reads to genomic features. Bioinformatics. 2013; 30:923–930. 24227677 10.1093/bioinformatics/btt656

[57] Love M.I., Huber W., Anders S. Moderated estimation of fold change and dispersion for RNA-seq data with DESeq2. Genome Biol. 2014; 15:550. 25516281 10.1186/s13059-014-0550-8 PMC4302049

[58] Yu G., Wang L.G., Han Y., He Q.Y. clusterProfiler: an R package for comparing biological themes among gene clusters. OMICS. 2012; 16:284–287. 22455463 10.1089/omi.2011.0118 PMC3339379

[59] Alpern D., Gardeux V., Russeil J., Mangeat B., Meireles-Filho A.C.A., Breysse R., Hacker D., Deplancke B. BRB-seq: ultra-affordable high-throughput transcriptomics enabled by bulk RNA barcoding and sequencing. Genome Biol. 2019; 20:71. 30999927 10.1186/s13059-019-1671-x PMC6474054

[60] Li H., Handsaker B., Wysoker A., Fennell T., Ruan J., Homer N., Marth G., Abecasis G., Durbin R. 1000 Genome Project Data Processing Subgroup The Sequence Alignment/Map format and SAMtools. Bioinformatics. 2009; 25:2078–2079. 19505943 10.1093/bioinformatics/btp352 PMC2723002

[61] Martin M. Cutadapt removes adapter sequences from high-throughput sequencing reads. EMBnet.journal. 2011; 17:10–12.

[62] Langmead B., Salzberg S.L. Fast gapped-read alignment with Bowtie 2. Nat. Methods. 2012; 9:357–359. 22388286 10.1038/nmeth.1923 PMC3322381

[63] Ramírez F., Ryan D.P., Grüning B., Bhardwaj V., Kilpert F., Richter A.S., Heyne S., Dündar F., Manke T. deepTools2: a next generation web server for deep-sequencing data analysis. Nucleic Acids Res. 2016; 44:160–165. 10.1093/nar/gkw257 PMC4987876 27079975

[64] Quinlan A.R., Hall I.M. BEDTools: a flexible suite of utilities for comparing genomic features. Bioinformatics. 2010; 26:841–842. 20110278 10.1093/bioinformatics/btq033 PMC2832824

[65] Chua E.Y.D., Vasudevan D., Davey G.E., Wu B., Davey C.A. The mechanics behind DNA sequence-dependent properties of the nucleosome. Nucleic Acids Res. 2012; 40:6338–6352. 22453276 10.1093/nar/gks261 PMC3401446

[66] Davey C.A., Sargent D.F., Luger K., Maeder A.W., Richmond T.J. Solvent mediated interactions in the structure of the nucleosome core particle at 1.9 Å resolution. J. Mol. Biol. 2002; 319:1097–1113. 12079350 10.1016/S0022-2836(02)00386-8

[67] Tachiwana H., Osakabe A., Shiga T., Miya Y., Kimura H., Kagawa W., Kurumizaka H. Structures of human nucleosomes containing major histone H3 variants. Acta Crystallogr. D Biol. Crystallogr. 2011; 67:578–583. 21636898 10.1107/S0907444911014818

[68] Kono H., Shirayama K., Arimura Y., Tachiwana H., Kurumizaka H. Two arginine residues suppress the flexibility of nucleosomal DNA in the canonical nucleosome core. PLoS One. 2015; 10:e0120635. 25786215 10.1371/journal.pone.0120635 PMC4365049

[69] Harada A., Maehara K., Sato Y., Konno D., Tachibana T., Kimura H., Ohkawa Y. Incorporation of histone H3.1 suppresses the lineage potential of skeletal muscle. Nucleic Acids Res. 2015; 43:775–86. 25539924 10.1093/nar/gku1346 PMC4333396

[70] Taguchi H., Xie Y., Horikoshi N., Maehara K., Harada A., Nogami J., Sato K., Arimura Y., Osakabe A., Kujirai T. et al. Crystal structure and characterization of novel human histone H3 variants, H3.6, H3.7 and H3.8. Biochemistry. 2017; 56:2184–2196. 28374988 10.1021/acs.biochem.6b01098

[71] van Velthoven C.T.J., de Morree A., Egner I.M., Brett J.O., Rando T.A. Transcriptional profiling of quiescent muscle stem cells *In Vivo*. Cell Rep. 2017; 21:1994–2004. 29141228 10.1016/j.celrep.2017.10.037 PMC5711481

[72] Tachiwana H., Kagawa W., Osakabe A., Kawaguchi K., Shiga T., Hayashi-Takanaka Y., Kimura H., Kurumizaka H. Structural basis of instability of the nucleosome containing a testis-specific histone variant, human H3T. Proc. Natl. Acad. Sci. U.S.A. 2010; 107:10454–10459. 20498094 10.1073/pnas.1003064107 PMC2890842

[73] Urahama T., Harada A., Maehara K., Horikoshi N., Sato K., Sato Y., Shiraishi K., Sugino N., Osakabe A., Tachiwana H. et al. Histone H3.5 forms an unstable nucleosome and accumulates around transcription start sites in human testis. Epigenet. Chromatin. 2016; 9:2. 10.1186/s13072-016-0051-y PMC4714512 26779285

[74] Kruger W., Peterson C.L., Sil A., Coburn C., Arents G., Moudrianakis E.N., Herskowitz I. Amino acid substitutions in the structured domains of histones H3 and H4 partially relieve the requirement of the yeast SWI/SNF complex for transcription. Genes Dev. 1995; 9:2770–2779. 7590252 10.1101/gad.9.22.2770

[75] Kurumizaka H., Wolffe A.P. Sin mutations of histone H3: influence on nucleosome core structure and function. Mol. Cell Biol. 1997; 17:6953–6969. 9372928 10.1128/mcb.17.12.6953 PMC232553

[76] Kujirai T., Ehara H., Fujino Y., Shirouzu M., Sekine S-I., Kurumizaka H. Structural basis of the nucleosome transition during RNA polymerase II passage. Science. 2018; 362:595–598. 30287617 10.1126/science.aau9904

[77] Ehara H., Kujirai T., Fujino Y., Shirouzu M., Kurumizaka H., Sekine S-I. Structural insight into nucleosome transcription by RNA polymerase II with elongation factors. Science. 2019; 363:744–747. 30733384 10.1126/science.aav8912

[78] Kujirai T., Horikoshi N., Sato K., Maehara K., Machida S., Osakabe A., Kimura H., Ohkawa Y., Kurumizaka H. Structure and function of human histone H3.Y nucleosome. Nucleic Acids Res. 2016; 44:6127–6141. 27016736 10.1093/nar/gkw202 PMC5291245

[79] Zink L.M., Delbarre E., Eberl H.C., Keilhauer E.C., Bönisch C., Pünzeler S., Bartkuhn M., Collas P., Mann M., Hake S.B. H3.Y discriminates between HIRA and DAXX chaperone complexes and reveals unexpected insights into human DAXX-H3.3-H4 binding and deposition requirements. Nucleic Acids Res. 2017; 45:5691–5706. 28334823 10.1093/nar/gkx131 PMC5449609

[80] Kujirai T., Horikoshi N., Xie Y., Taguchi H., Kurumizaka H. Identification of the amino acid residues responsible for stable nucleosome formation by histone H3.Y. Nucleus. 2017; 8:239–248. 28118111 10.1080/19491034.2016.1277303 PMC5499910

[81] Conde e Silva N., Black B.E., Sivolob A., Filipski J., Cleveland D.W., Prunell A. CENP-A-containing nucleosomes: easier disassembly versus exclusive centromeric localization. J. Mol. Biol. 2007; 370:555–573. 17524417 10.1016/j.jmb.2007.04.064

[82] Tachiwana H., Kagawa W., Shiga T., Osakabe A., Miya Y., Saito K., Hayashi-Takanaka Y., Oda T., Sato M., Park S.Y. et al. Crystal structure of the human centromeric nucleosome containing CENP-A. Nature. 2011; 476:232–235. 21743476 10.1038/nature10258

[83] Boopathi R., Danev R., Khoshouei M., Kale S., Nahata S., Ramos L., Angelov D., Dimitrov S., Hamiche A., Petosa C. et al. Phase-plate cryo-EM structure of the Widom 601 CENP-A nucleosome core particle reveals differential flexibility of the DNA ends. Nucleic Acids Res. 2020; 48:5735–5748. 32313946 10.1093/nar/gkaa246 PMC7261176

[84] Roulland Y., Ouararhni K., Naidenov M., Ramos L., Shuaib M., Syed S.H., Lone I.N., Boopathi R., Fontaine E., Papai G. et al. The flexible ends of CENP-A nucleosome are required for mitotic fidelity. Mol. Cell. 2016; 63:674–685. 27499292 10.1016/j.molcel.2016.06.023

[85] Arimura Y., Kimura H., Oda T., Sato K., Osakabe A., Tachiwana H., Sato Y., Kinugasa Y., Ikura T., Sugiyama M. et al. Structural basis of a nucleosome containing histone H2A.B/H2A.Bbd that transiently associates with reorganized chromatin. Sci. Rep. 2013; 3:3510. 24336483 10.1038/srep03510 PMC3863819

[86] Sugiyama M., Arimura Y., Shirayama K., Fujita R., Oba Y., Sato N., Inoue R., Oda T., Sato M., Heenan R.K. et al. Distinct features of the histone core structure in nucleosomes containing the histone H2A.B variant. Biophys. J. 2014; 106:2206–2213. 24853749 10.1016/j.bpj.2014.04.007 PMC4052288

[87] Hirano R., Arimura Y., Kujirai T., Shibata M., Okuda A., Morishima K., Inoue R., Sugiyama M., Kurumizaka H. Histone variant H2A.B-H2B dimers are exchanged with canonical H2A–H2B in the nucleosome. Commun. Biol. 2021; 4:191. 33580188 10.1038/s42003-021-01707-z PMC7881002

[88] Hirano R., Kujirai T., Negishi L., Kurumizaka H. Biochemical characterization of the placeholder nucleosome for DNA hypomethylation maintenance. Biochem. Biophys. Rep. 2019; 18:100634. 31008378 10.1016/j.bbrep.2019.100634 PMC6458450

[89] Syed S., Boulard M., Shukla M.S., Gautier T., Travers A., Bednar J., Faivre-Moskalenko C., Dimitrov S., Angelov D. The incorporation of the novel histone variant H2AL2 confers unusual structural and functional properties of the nucleosome. Nucleic Acids Res. 2009; 37:4684–4695. 19506029 10.1093/nar/gkp473 PMC2724287

[90] Barral S., Morozumi Y., Tanaka H., Montellier E., Govin J., de Dieuleveult M., Charbonnier G., Couté Y., Puthier D., Buchou T. et al. Histone variant H2AL2 guides transition protein-dependent protamine assembly in male germ cells. Mol. Cell. 2017; 66:89–101. 28366643 10.1016/j.molcel.2017.02.025

[91] Takizawa Y., Ho C.H., Tachiwana H., Matsunami H., Kobayashi W., Suzuki M., Arimura Y., Hori T., Fukagawa T., Ohi M.D. et al. Cryo-EM structures of centromeric tri-nucleosomes containing a central CENP - a nucleosome. Structure. 2020; 28:44–53. 31711756 10.1016/j.str.2019.10.016

[92] Shukla M.S., Syed S.H., Goutte-Gattat D., Richard J.L., Montel F., Hamiche A., Travers A., Faivre-Moskalenko C., Bednar J., Hayes J.J. et al. The docking domain of histone H2A is required for H1 binding and RSC-mediated nucleosome remodeling. Nucleic Acids Res. 2011; 39:2559–2570. 21131284 10.1093/nar/gkq1174 PMC3074127

[93] Bannister A.J., Kouzarides T. Regulation of chromatin by histone modifications. Cell Res. 2011; 21:381–395. 21321607 10.1038/cr.2011.22 PMC3193420

[94] Doyen C.M., Montel F., Gautier T., Menoni H., Claudet C., Delacour-Larose M., Angelov D., Hamiche A., Bednar J., Faivre-Moskalenko C. et al. Dissection of the unusual structural and functional properties of the variant H2A.Bbd nucleosome. EMBO J. 2006; 25:4234–4244. 16957777 10.1038/sj.emboj.7601310 PMC1570437

[95] Arimura Y., Kimura H., Oda T., Sato K., Osakabe A., Tachiwana H., Sato Y., Kinugasa Y., Ikura T., Sugiyama M. et al. Structural basis of a nucleosome containing histone H2A.B/H2A.Bbd that transiently associates with reorganized chromatin. Scientific Rep. 2013; 3:3510. 10.1038/srep03510 PMC3863819 24336483

[96] Gautier T., Abbott D.W., Molla A., Verdel A., Ausio J., Dimitrov S. Histone variant H2ABbd confers lower stability to the nucleosome. EMBO Rep. 2004; 5:715–720. 15192699 10.1038/sj.embor.7400182 PMC1299093

[97] Zhou M., Dai L., Li C., Shi L., Huang Y., Guo Z., Wu F., Zhu P., Zhou Z. Structural basis of nucleosome dynamics modulation by histone variants H2A.B and H2A.Z.2.2. EMBO J. 2021; 40:e105907. 33073403 10.15252/embj.2020105907 PMC7780145

