# Supplementary material for: Unusual nucleosome formation and transcriptome influence by the histone H3mm18 variant
Source: Nucleic Acids Res. 2021 Dec 21;50(1):72–91. doi: 10.1093/nar/gkab1137 (PMC8855299; doi:10.1093/nar/gkab1137)
Supplement: gkab1137_Supplemental_File [file gkab1137_supplemental_file.pdf]

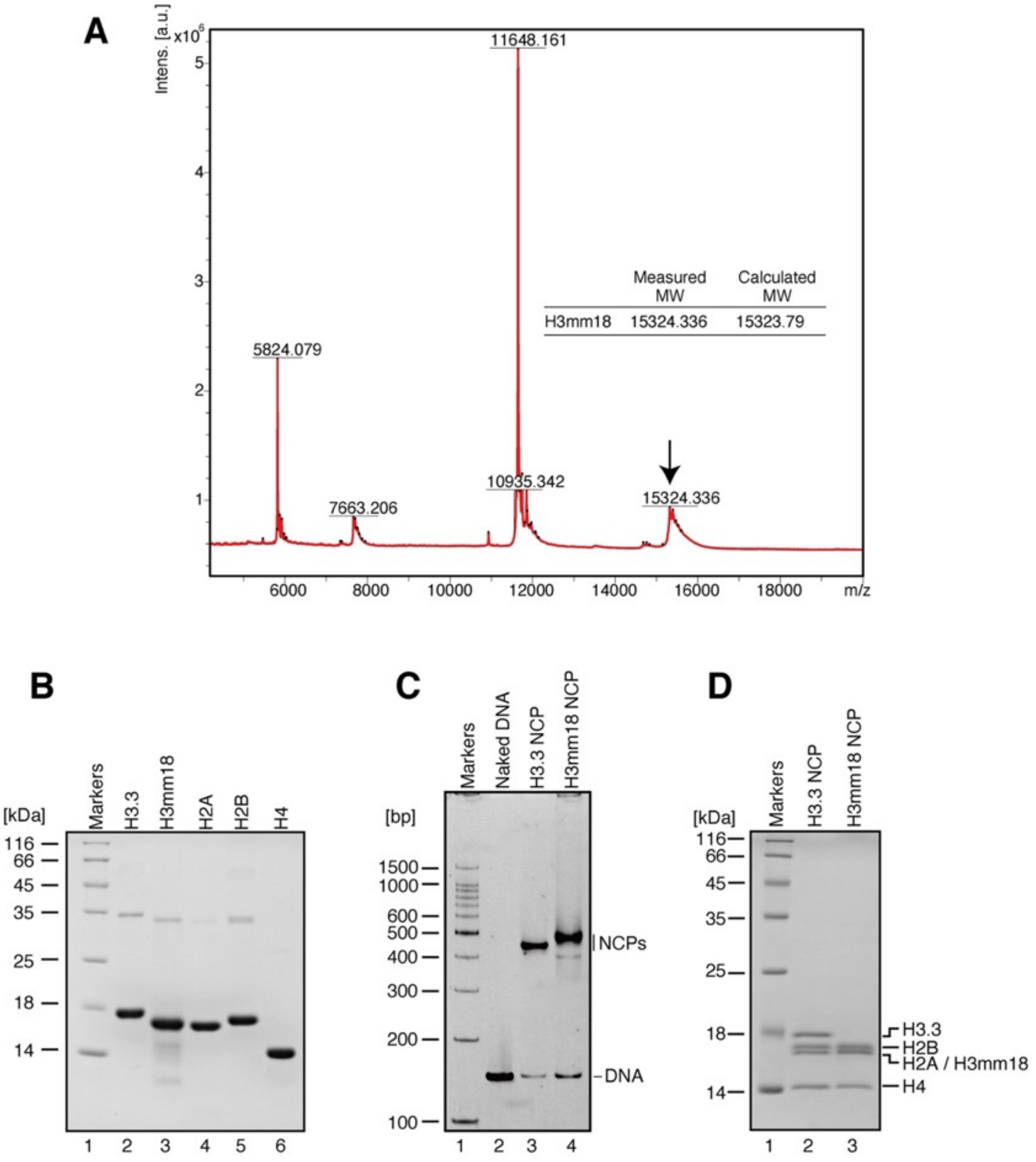

Supplementary Figure S1. Purification of the H3mm18 NCP. (A) The molecular mass of the prepared H3mm18-H4 complex was determined by a MALDI-TOF MS analysis. The arrow indicates the peak corresponding to H3mm18. The measured and calculated molecular weights (MW) of H3mm18 are shown. The calculated MW was estimated using Expasy compute MW (https://web.expasy.org/peptide_mass/). (B) The purified mouse H3.3, H3mm18, H2A, H2B, and H4 were analyzed by SDS-PAGE with Coomassie Brilliant Blue (CBB) staining. (C) The purified NCPs containing H3.3 or H3mm18 were analyzed by native-PAGE with ethidium bromide staining. (D) The purified NCPs were analyzed by SDS-PAGE with CBB staining.

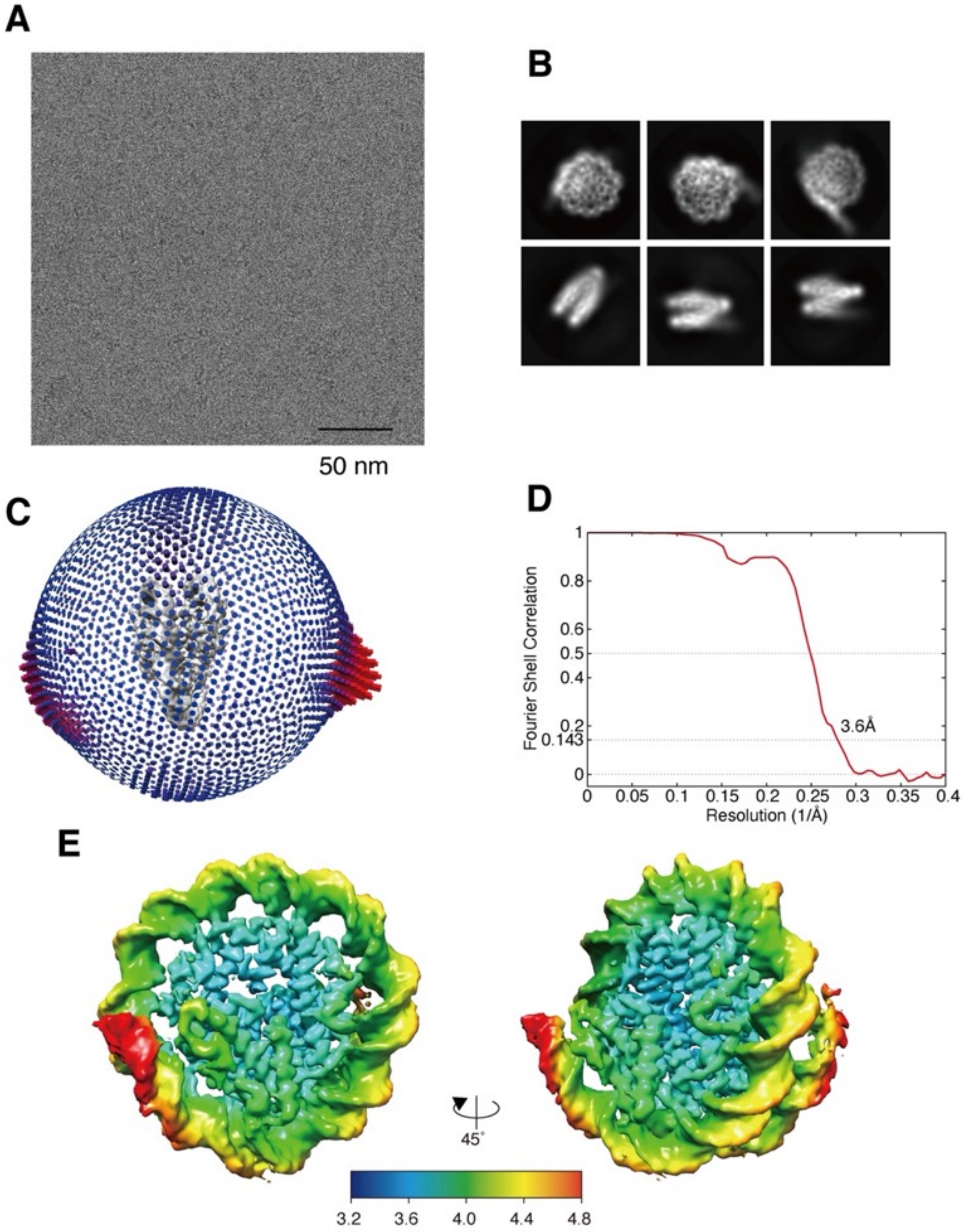

Supplementary Figure S2. Cryo-EM analysis of the H3mm18 NCP. (A) A representative micrograph of the H3mm18 NCP. (B) Two-dimensional class average of the H3mm18 NCP structure. (C) Euler angular distribution of the H3mm18 NCP structure. (D) Fourier Shell Correlation (FSC) curve of the H3mm18 NCP structure. The resolution of the H3mm18 NCP structure was estimated to be 3.6 Å by an FSC = 0.143. (E) Local resolution map of the H3mm18 NCP structure.

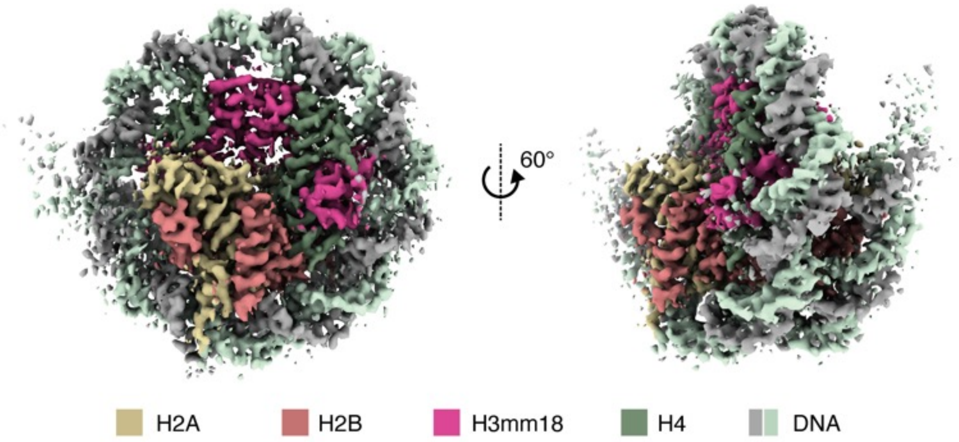

Supplementary Figure S3. Cryo-EM density map of the non-crosslinked H3mm18 NCP stabilized by PL2-6 scFv. Histones H3mm18, H2A, H2B, and H4 are colored pink, yellow, salmon pink, and green, respectively. DNA is colored gray and light green. To enhance the clarity of the H3mm18 NCP structure, the cryo-EM density corresponding to the PL2-6 scFv was subtracted.

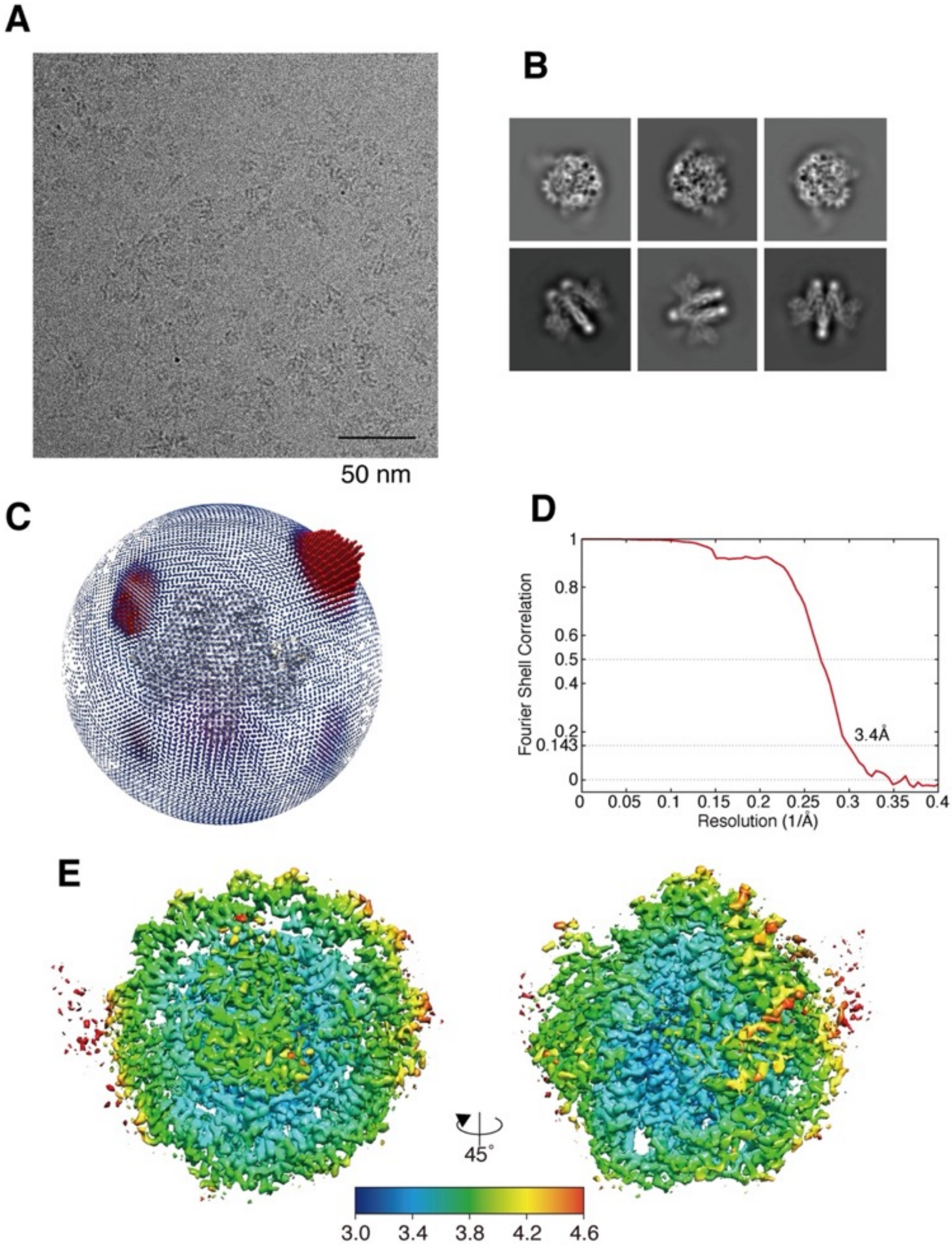

Supplementary Figure S4. Cryo-EM analysis of the non-crosslinked H3mm18 NCP with PL2-6 scFv. (A) Representative micrograph of the non-crosslinked H3mm18 NCP complexed with the PL2-6 scFv. (B) Two-dimensional class average of the H3mm18 NCP-PL2-6 scFv complex. (C) Euler angular distribution of the H3mm18 NCP-PL2-6 scFv complex. (D) Fourier Shell Correlation (FSC) curve of the H3mm18 NCP-PL2-6 scFv complex. The resolution of the H3mm18 NCP-PL2-6 scFv complex was estimated to be 3.4 Å by an FSC = 0.143. (E) Local resolution map of the H3mm18 NCP-PL2-6 scFv complex.

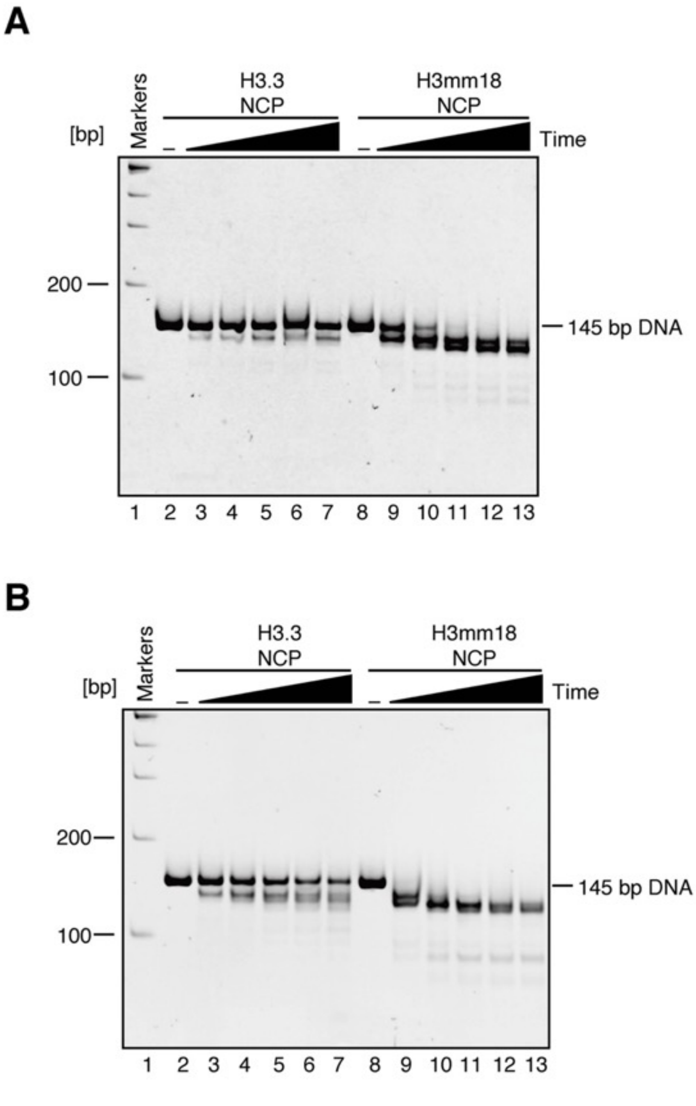

Supplementary Figure S5. (A), (B) Repeated MNase treatment assays, as shown in Figure 3B.

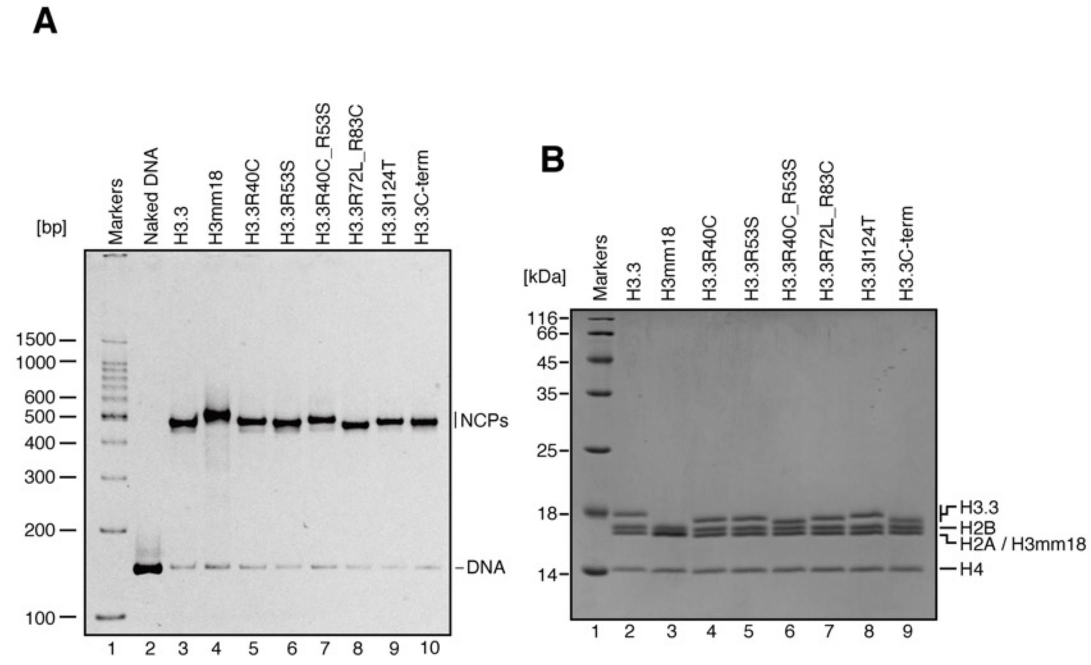

Supplementary Figure S6. Purification of the NCPs containing H3.3 mutants. (A) The purified NCPs containing H3.3, H3mm18, or H3.3 mutant were analyzed by native-PAGE with ethidium bromide staining. (B) The purified NCPs were analyzed by SDS-PAGE with CBB staining.

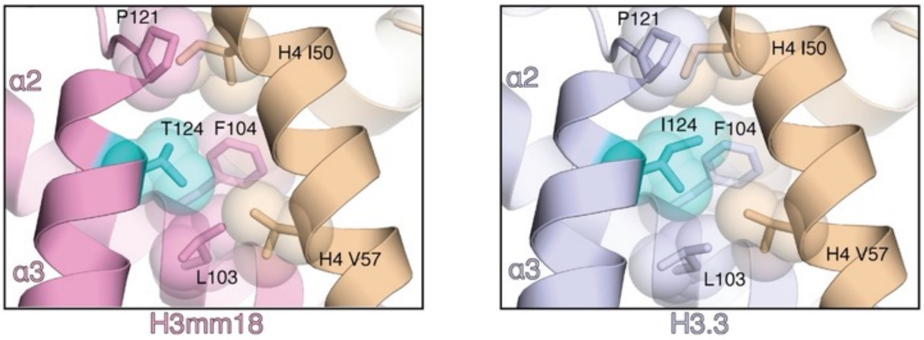

Supplementary Figure S7. Structural comparison around position 124 in the H3mm18 NCP (left panel) and H3.3 NCP (right panel). In the left panel, the H3mm18 Thr124 residue is shown in cyan with its side chain. In the right panel, the H3 Ile124 residue is shown in cyan with its side chain. The atoms located between H3mm18 (pink) or H3 (light blue) and H4 (beige) are presented as spheres.

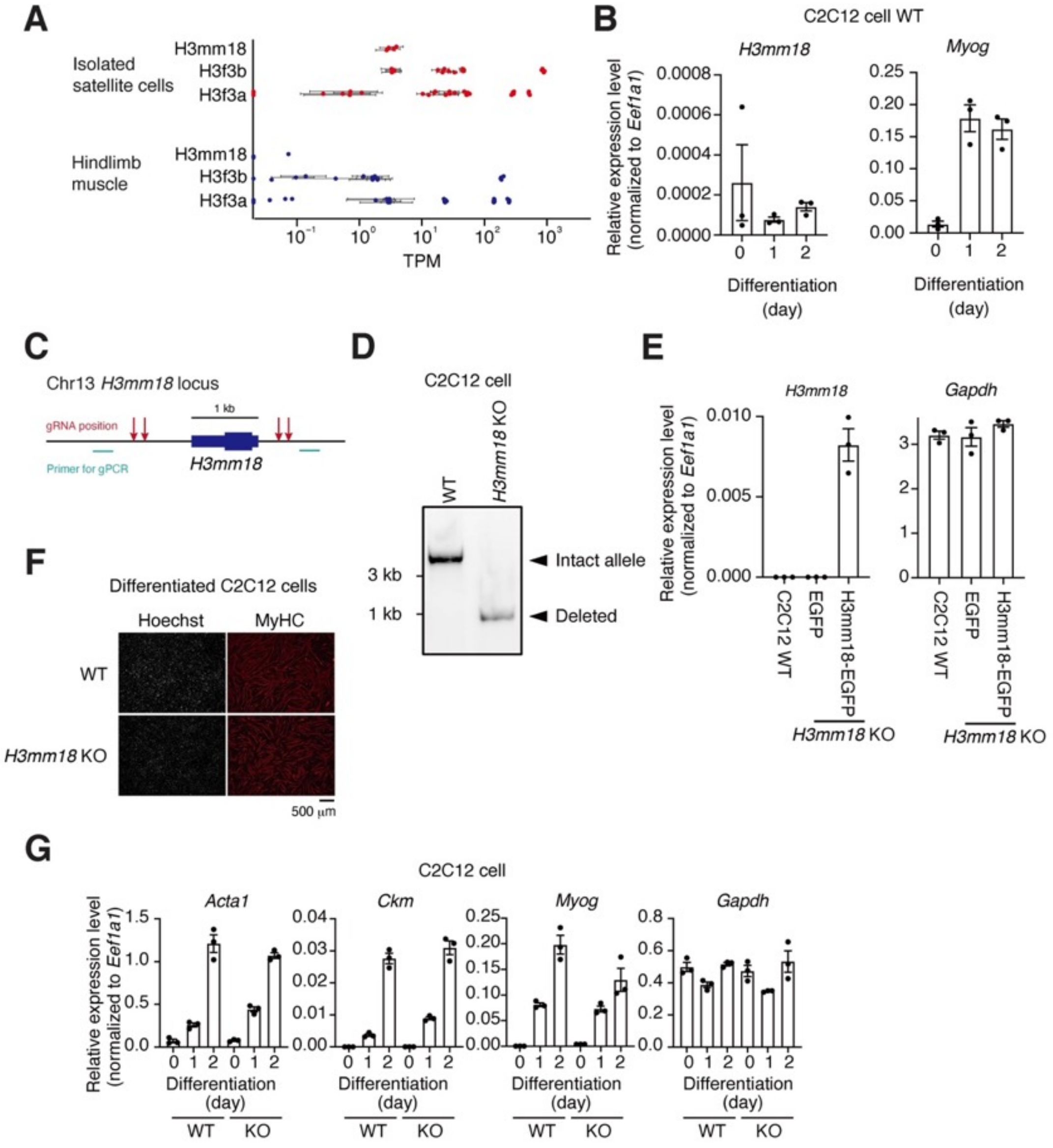

Supplementary Figure S8. Evaluation of endogenous H3mm18 expression in C2C12 cells. (A) Endogenous expression of the *H3mm18* gene in isolated satellite cells and differentiated muscle. (B) qRT-PCR analysis of H3mm18 during differentiation. RNAs were extracted from undifferentiated and differentiated WT C2C12 cells. (C) Schematic illustration of the H3mm18 deletion using the CRISPR/Cas9 system. (D) Agarose gel electrophoresis for genotyping. Genomic DNAs were prepared from WT and H3mm18 knocked-out C2C12 cells. PCR was performed with a primer pair targeting the *H3mm18* locus. (E) qRT-PCR analysis of H3mm18. RNAs were extracted from C2C12 WT and KO cells expressing EGFP or EGFP-H3mm18. (F) ICC analysis of differentiated C2C12 cells (WT and H3mm18 KO cells). (G) qRT-PCR analysis of C2C12 cells (WT and H3mm18 KO cells) during differentiation.

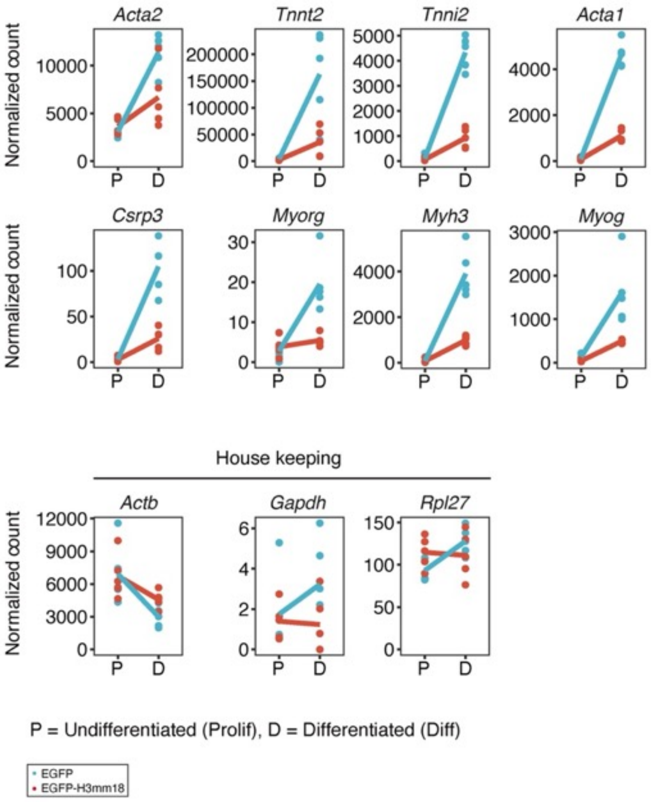

Supplementary Figure S9. Gene expression patterns of C2C12 cells during differentiation. Expression patterns of representative myogenic and housekeeping genes corresponding to Figure 7C are shown.

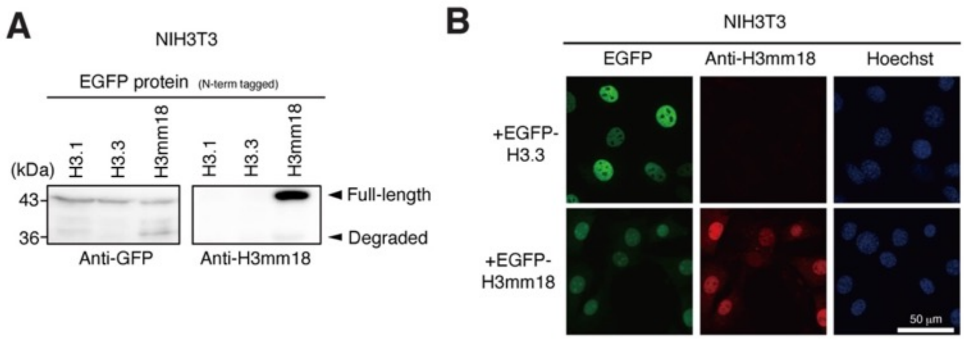

Supplementary Figure S10. Validation of the anti-H3mm18 antibody. (A) Immunoblot analysis to validate the H3mm18 antibody, using NIH3T3 cells expressing EGFP-tagged H3.1, H3.3 or H3mm18. (B) ICC analysis of the H3mm18 antibody. NIH3T3 cells expressing EGFP-tagged H3.3 or H3mm18 were used.
